# Altered brain functional network dynamics in classic trigeminal neuralgia: a resting-state functional magnetic resonance imaging study

**DOI:** 10.1186/s10194-021-01354-z

**Published:** 2021-12-11

**Authors:** Pengfei Zhang, Yanli Jiang, Guangyao Liu, Jiao Han, Jun Wang, Laiyang Ma, Wanjun Hu, Jing Zhang

**Affiliations:** 1 Second Clinical School, Lanzhou University, Lanzhou, 730000 China; 2 Department of Magnetic Resonance, Lanzhou University Second Hospital, Lanzhou, 730000 China; 3 Gansu Province Clinical Research Center for Functional and Molecular Imaging, Cuiyingmen No.82, Chengguan District, Lanzhou, 730030 P. R. China

**Keywords:** Trigeminal neuralgia, Chronic pain, Resting-state functional MRI, Dynamic functional connectivity, Dynamic pain connectome

## Abstract

**Background:**

Accumulating studies have indicated a wide range of brain alterations with respect to the structure and function of classic trigeminal neuralgia (CTN). Given the dynamic nature of pain experience, the exploration of temporal fluctuations in interregional activity covariance may enhance the understanding of pain processes in the brain. The present study aimed to characterize the temporal features of functional connectivity (FC) states as well as topological alteration in CTN.

**Methods:**

Resting-state functional magnetic resonance imaging and three-dimensional T1-weighted images were obtained from 41 CTN patients and 43 matched healthy controls (HCs). After group independent component analysis, sliding window based dynamic functional network connectivity (dFNC) analysis was applied to investigate specific FC states and related temporal properties. Then, the dynamics of the whole brain topological organization were estimated by calculating the coefficient of variation of graph-theoretical properties. Further correlation analyses were performed between all these measurements and clinical data.

**Results:**

Two distinct states were identified. Of these, the state 2, characterized by complicated coupling between default mode network (DMN) and cognitive control network (CC) and tight connections within DMN, was expressed more in CTN patients and presented as increased fractional windows and dwell time. Moreover, patients switched less frequently between states than HCs. Regarding the dynamic topological analysis, disruptions in global graph-theoretical properties (including network efficiency and small-worldness) were observed in patients, coupled with decreased variability in nodal efficiency of anterior cingulate cortex (ACC) in the salience network (SN) and the thalamus and caudate nucleus in the subcortical network (SC). The variation of topological properties showed negative correlation with disease duration and attack frequency.

**Conclusions:**

The present study indicated disrupted flexibility of brain topological organization under persistent noxious stimulation and further highlighted the important role of “dynamic pain connectome” regions (including DMN/CC/SN) in the pathophysiology of CTN from the temporal fluctuation aspect. Additionally, the findings provided supplementary evidence for current knowledge about the aberrant cortical-subcortical interaction in pain development.

**Supplementary Information:**

The online version contains supplementary material available at 10.1186/s10194-021-01354-z.

## Background

Classic trigeminal neuralgia (CTN) is a chronic pain disorder, characterized by unilateral paroxysmal electric shock-like or stabbing painful affliction of the face, which is limited to the trigeminal territory [1]. With disease progression, episodes of pain occur more frequently and sustainably, which seriously impacts the patient’s physical and psychological health [2]. CTN has been generally attributed to the nerve vessel conflict (NVC) at the nerve root entry zone [3]. However, accumulating evidence suggests that the involvement of centrally mediated facilitation of pain processing or reduced descending inhibitory mechanisms play an important part in the CTN pathogenesis [4, 5]. Therefore, the central nervous system mechanism of CTN has now become a research focus.

To noninvasively map brain activity, resting state functional magnetic resonance imaging (rs-fMRI) has been widely used which has further revealed several critical brain regions of CTN, including the default mode-, the salience-, the subcortical- and the sensorimotor networks [6–11]. These regions are closely related to pain perception, modulation, the cognitive-affective interaction, and motor control [12]. Aberrant information transfer within and across brain networks and maladaptive brain plasticity in CTN patients may underpin disease pathogenesis [7]. Notably, the aforementioned rs-fMRI studies assumed that the brain connectivity pattern is stationary during scan sessions. However, actual brain activity is highly dynamic and condition-dependent [13]. Correspondingly, recent findings suggested that resting-state functional connectivity (rsFC) can fluctuate spontaneously on multiple time scales [14, 15].

As an intrinsically dynamic experience encoded by “pain connectome ”[16], pain fluctuates spontaneously over time and is influenced by many dynamic factors [17–19]. It is the uncovering of dynamics in FC across time scales and its interaction with external factors that helps improve the understanding of the central pain processes [20]. Recently, one dynamic regional homogeneity study detected temporal alteration about spontaneous neural activity in TN patients [21], but did not focus on changes in dynamic FC. However, dynamic functional network connectivity (dFNC) analysis can not only provide time-varying information of FC between resting-state connectivity networks (RSNs )[13], but also capture reproducible connectivity states and calculate temporal properties. dFNC has been applied in chronic pain studies such as those on migraine [22, 23], low back pain [24], and primary dysmenorrhea [25], and has been indicated as a useful potential biomarkers of pain and bee effective to provide insights on disease pathogenesis [26]. However, the exploration of changes of the whole-brain dFNC pattern in CTN is still limited.

Previously, some rs-fMRI studies using graph theory have attempted to characterize the global brain modularity as well as key nodal features in CTN to elucidate the reorganization processes of brain networks [8, 27]. Given that the FC between brain regions is constrained by the brain topological organization [28], aberrant dFNC pattern may be additionally accompanied by altered dynamic topological properties.

In this study, we applied sliding window approach based on rs-fMRI to investigate the time-varying characteristics of CTN patients. Specifically, CTN specific dFNC state was identified and diverse temporal properties were calculated to assess brain dynamics. Moreover, dynamic graph theoretic analysis was used to investigate the temporal changes of global and nodal topological organization. We hypothesized that CTN patients would show altered temporal properties coupled with aberrant network topological dynamics, which would be correlated with clinical characteristics.

## Methods

### Participants and clinical characteristics assessment

In total, this study included 43 patients with CTN matched for age, sex, and education with 45 healthy controls (HCs). All patients were recruited from Lanzhou University Second Hospital and diagnosed according to the International Classification of Headache Disorders (ICDH-III )[2] by two experienced neurologists. NVC was demonstrated for all patients on either MRI or during surgery. The inclusion criteria were as follows: (1) duration of disease was > 1 years; (2) unilateral pain in the area innervated by one or more branches of the trigeminal nerve; (3) paroxysmal pain, described as electric shock-like, shooting or stabbing experience, activated by trigger factors or in the trigger areas; and (4) absence of obvious sensory deficits. The exclusion criteria were as follow: (1) CTN with concomitant continuous pain; (2) surgical history, especially microvascular decompression (MVD) for CTN, or a history of head trauma; (3) presence of any other pain disorders or neuropsychiatric disease, (4) metal implants in the body, particularly metallic fixed dentures; and (5) abnormal MR manifestation (including severe white matter lesions with Fazekas grade 3 and evidence of multiple sclerosis or space-occupying lesions that indicate secondary TN )[9]. All CTN patients all received medical treatment, of which carbamazepine was the most common, followed by mecobalamin. Details about medicine use history can be seen in Table 1. No controls about patients’ treatment were taken. The research was approved by the Ethics Committee of Lanzhou University Second Hospital. According to the Declaration of Helsinki, written consent was obtained from every participant after the study details were explained to the patients.

**Table 1:** Demographic and clinical characteristics of participants

	Patients with CTN	Healthy controls	χ^2^/*t* value	*P*-value
Sex (female/male)	23/18	24/19	0.001	0.979
Age, y	56.34 ± 10.50	53.40 ± 9.73	−1.344	0.186
Education, y	11.66 ± 2.33	12.07 ± 2.24	0.825	0.412
Duration of disease, y	5.79 ± 4.68	NA	NA	NA
Attack frequency (times per day)	7.41 ± 3.91	NA	NA	NA
Score of VAS	6.41 ± 0.91	NA	NA	NA
Attack side	Right (24); Left (17)	NA	NA	NA
Medication	Carbamazepine (33)/ Mecobalamin (5)/ None (3)	NA	NA	NA

Values were displayed as mean ± SD (range). *p* value of sex was calculated by chi-square test and *p* values of age, education were obtained by independent-samples t-test. *CTN*, classic trigeminal neuralgia; *HC*, healthy controls; *VAS*, visual analogue scale

The pain intensity of CTN patients was recorded with visual analogue scale (VAS) ranging from 0 (no pain) to 10 (worst imaginable pain). Patients were required to rate their pain intensity in the last 7 days by marking on the 100-mm questionnaire line, which was then averaged to obtain the weekly score. All questionnaire assessment was performed under the supervision of experimenters.

### MRI data acquisition

Structural MRI and rs-fMRI data were acquired on a 3.0 T Siemens Verio MRI system (Siemens Medical System, Erlangen, Germany) with an 8-channel head coil. During scanning, participants were instructed to stay awake and relaxed but to keep their eyes closed, with earplugs and foam padding used to attenuate noise and reduce head motion. High-resolution three-dimensional structural images were acquired using sagittal Magnetization Prepared Rapid Gradient echo sequence (field of view: 256 × 256 mm; matrix: 256 × 256; time of repetition = 1900 ms; time of echo = 2.93 ms; resolution = 1 × 1 mm; flip angle = 9°). The rs-fMRI images were acquired via an echo-planar imaging (EPI) sequence (180 volumes, 36 contiguous slices, FOV, 192 × 192 mm, matrix: 64 × 64, spatial resolution = 3 × 3 × 3 mm; TR = 2000 ms; TE =30 ms; flip angle = 90°). An experienced radiologist inspected the previous MR images of these participants to ensure that each patient was free of abnormalities as described in above exclusion criteria (5).

### fMRI data preprocessing and head motion analysis

The rs-fMRI data were preprocessed with the toolbox for Data Processing & Analysis of Brain Imaging (DPABI, http://rfmri.org/dpabi )[29]. The first 10 volumes of the functional images were discarded. The remaining volumes underwent slice-time correction, and were then realigned to correct the motion between time points, wherein, head motion parameters were computed by estimating the translation in each direction and the angular rotation on each axis for each volume. As a result, the participants with mean framewise displacement (FD) (Jenkinson) > 0.2 mm or head displacement > 1.5 mm, maximum rotation > 1.5° were excluded from the analysis. According to this exclusion criterion, two subjects each from the HC and CTN group were excluded. No significant intergroup differences were found in FD (*t* = 1.09, *p* = 0.28). The individuals’ fMRI data were co-registered to their structural images, followed by segmentation of the gray matter (GM), white matter (WM), and cerebrospinal fluid (CSF), and normalization to the Montreal Neurological Institute (MNI) space. The normalized images were spatially smoothed with a 6-mm full-width at half-maximum Gaussian kernel.

### Group independent component analysis (GICA) analysis and identification of independent components

After preprocessing, fMRI images with 170 volumes underwent GICA to be decomposed into different RSNs by using the Group ICA of fMRI Toolbox software (version 4.0b; mialab.mrn.org/software/gift/ )[30]. Two data reduction steps were performed in the principal component analysis [14]. First, we reduced the individuals’ data into 120 principal components, which preserved > 99% of the variance. Next, we concatenated the reduced data of all participants across time and further reduced the data to 100 principal components using an expectation maximization algorithm [31]. The reliability and stability of the infomax ICA algorithm [32] was ensured by iterating 20 times in the ICASSO implemented in GIFT [33] and using the most central run to reconstruct subject-specific time courses and spatial maps of each IC using the GICA back reconstruction algorithm [34]. The group ICs of the 20 runs were clustered to estimate their reliability, values > 0.8 were selected [35]. By using one sample t-test across all subjects and for each IC, the t-map of ICs was obtained with a threshold of *t* > mean (*μ*) + 4SD (*σ* )[36]. Details about labels and spatial maps of each IC are presented in Fig. S2, and the peak coordinates of ICs are shown in Table S1.

**Fig. 1:**
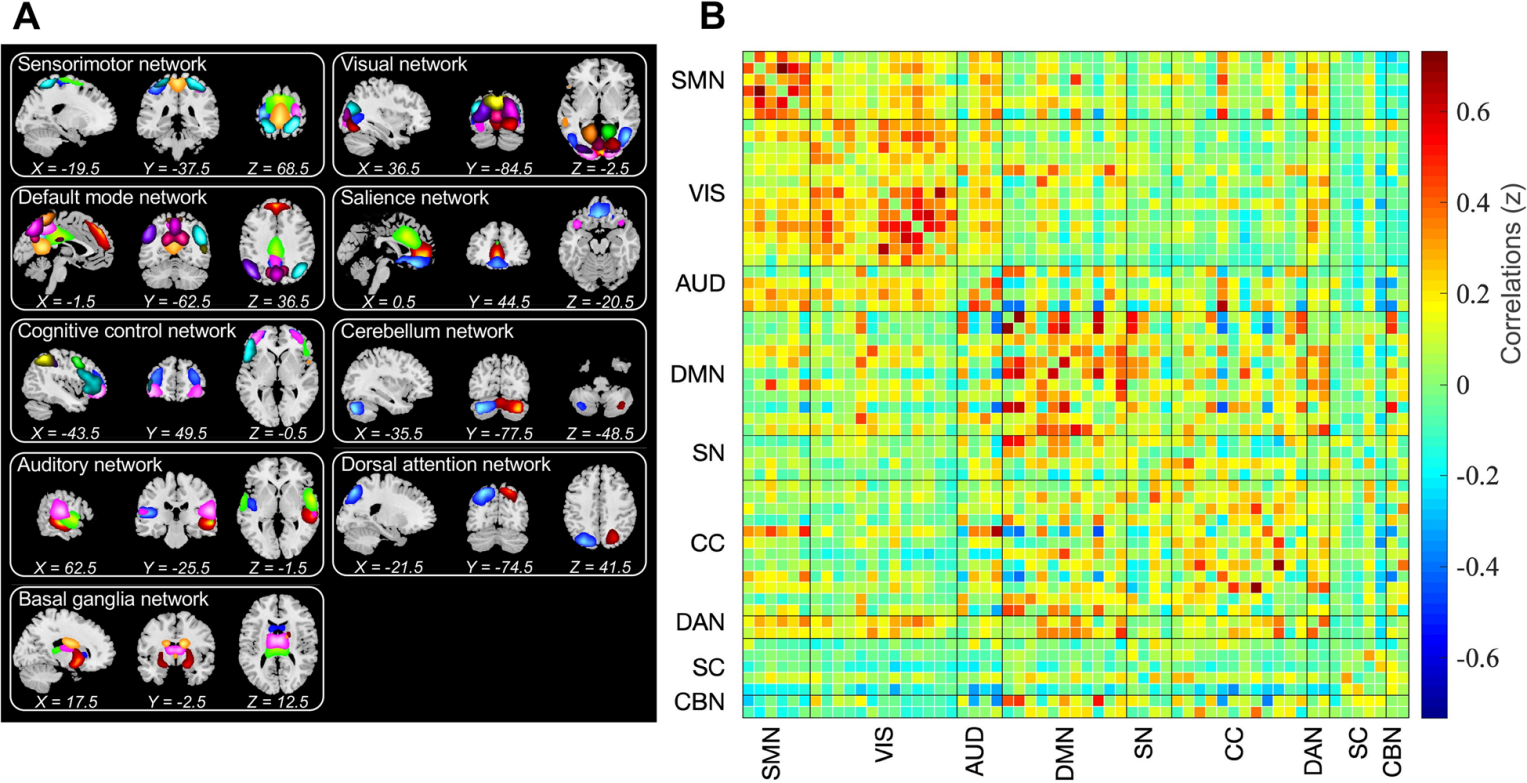
The spatial maps of 59 independent components and corresponding static functional network connectivity matrix. (A) 59 ICs were identified and grouped into 9 functional network. (B) sFC between the whole time courses of selected ICs were calculated and averaged over subjects. Color bar represents correlation values, i.e., Fisher’s z-transformed Pearson correlation coefficient. SMN, sensorimotor network; VIS, visual network; AUD, auditory network; DMN, default mode network; SN, salience network; CC, cognitive control network; DAN, dorsal attention network; SC, subcortical network; CBN, cerebellar network; sFC, static functional connectivity

We identified 59 ICs from 100 ICs based on the following evaluation criteria: (1) IC should exhibit peak activations in grey matter; (2) low spatial overlap with known vascular, ventricular, motion, and susceptibility artifacts; and (3) IC should have time courses dominated by low-frequency fluctuations (ratio of powers below 0.1 Hz to 0.15–0.25 Hz in the spectrum )[37]. All 59 ICs were then sorted into nine different RSNs according to the spatial correlation values between their spatial maps and atlas used in previous studies [14, 23, 38–40] (Fig. 1A). Afterwards, additional postprocessing was applied to the time courses of 59 ICs as described in Allen et al.’s study [14], including detrending, despiking using AFNI’s 3dDespike algorithm, filtering using a fifth-order Butterworth filter with a 0.15-Hz high frequency cut-off, and finally regressing out the movement parameters.

### dFNC estimation

We computed dFNC between the time courses (170 time points) of ICs using a sliding window approach, which was performed using the DFNC network toolbox in GIFT. A window size with 20-TR (40 s) was chosen, because previous studies suggested that FC fluctuations at resting-state would be captured with windows of 30 ~ 60s [41]. We used a tapered window in steps of 1 TR, which was obtained by convolving a rectangle with a Gaussian (*σ* = 3) function to localize the dataset at each time point. Finally, a total of 150 windows were obtained and 59 × 59 pairwise FC matrices by regularized precision matrix (inverse covariance matrix) [42] were computed in every window. The L1 norm penalty was imposed in the Graphical LASSO framework with 100 repetitions to promote sparsity in estimations [43]. With Fisher’s z-transformation, the correlation values of pairwise functional matrices were converted to z-values to improve normality and comparability and then residualized with nuisance variables, including age and sex [36].

### State clustering analysis

To assess the dFNC patterns that reoccur over time, k-means clustering was performed on all FC matrices for all participants. The k-means clustering algorithm was iterated 100 times with L1 distance (Manhattan distance) function to estimate the similarity between matrices [44]. Later, the analysis for cluster number validity was made and the optimal number of k was determined as 2, based on the silhouette criterion [45], which was computed as a ratio of the similarity between windows in the same cluster compared to similarity with windows in a different cluster (refer to supplementary methods for details of k-means clustering processes). In the next 100 clustering iterations, k = 2 was maintained. Eventually, we obtained two recurring FC states, of which cluster centroids were determined as the median of all matrices allocated to that state over time. The subject-specific centroids of each state were calculated similarly as the median value. Further, the subject-specific centroids belong to each group were averaged to obtain the group-specific centroids for better visualization of group comparison patterns [39].

To describe the characteristics of the two cluster states, we mainly focused on the degree of global modularity. Modularity is a valuable measurement from graph theory to interpret the dFNC states, because it evaluates both functional integration and segregation of networks [46]. Thus, we calculated the modularity index Q for each state by using a normal Louvain community detection algorithm in the Brain Connectivity Toolbox (www.brain-connectivity-toolbox.net/). A larger Q represents a higher tendency of assigning ICs into different modules [47]. Furthermore, the consensus clustering approach was used to solve the stochastic problem of the Louvain algorithm (refer to supplementary methods for details of modularity analysis) [48, 49].

We calculated four temporal properties: fractional windows, mean dwell time, number of transitions and transition likelihood. The fractional window is calculated as the proportion of time spent in each state as measured by percentage. The mean dwell time represents the average duration of time intervals an individual spent in each state, which was calculated by averaging the number of consecutive windows belonging to one state before switching to another. The number of transitions represents the switching times between states, which estimates the brain flexibility. The transition likelihood, represents the percentage of switching probability between states. For between-group comparison of different properties, nonparametric permutation tests (10,000 repetition) were used to assess differences in all those temporal properties mentioned above, treating age and sex as covariates. False discovery rate (FDR) correction was applied for fractional windows and mean dwell time.

For the purpose of evaluating the consistency and validity of the k-means clustering at different window sizes, we repeated the dFNC states analysis with 16-TR (32 s) and 24-TR (48 s). Pearson’s correlation coefficients between the cluster centroids under different window sizes was used to represent similarity and help to find the states consistent with the primary analysis [47].

### Dynamic topological analysis

We applied a graph theory approach to obtain topological metrics across all sliding windows of all subjects using GRETNA software (www.nitrc.org/projects/gretna), to observe the variability of topological organization of the functional connectivity network. Based on the framework of graph theory, we defined the 59 ICs as functionally independent nodes with FC between pairs of ICs as edges. At first, FC matrices of all windows were binarized with a series of sparsity thresholds, where edges larger than the threshold were designated as 1 and those smaller than the threshold were designated as 0. Only positive FC values were considered. Sparsity was defined as the ratio of the number of existing edges divided by the maximum possible number of edges in a network. Referring to previous studies [23, 28], we determined thresholds that ranged from 0.10 to 0.35 (with an interval of 0.01) for further analyses.

Next, we calculated both global and regional network properties in a series of adjacent matrices for all participants. The former included: (1) measures of global (E_g_) and local network efficiency (E_loc_); and (2) small-world global metrics of clustering coefficient (*C*), characteristic path length (*L*), small-worldness (*σ*), normalized clustering coefficient (*γ*), and normalized characteristic path length (*λ*); and the later was nodal efficiency. Given that it was widely used in previous studies, an area under the curve (AUC) approach was chosen to avoid the specific selection of a threshold [23]. The detailed interpretation of topological properties is listed in Table S2. To better characterize the temporal variation of those measurements, we also computed the coefficient of variation (CV) of AUC of network parameters as performed by Luo et al. [28], where CV was calculated as the mean divided by the standard deviation (SD) across all sliding windows.

The nonparametric permutation approach (10,000 iterations) was used again to test for dynamic topological property differences in the AUC of each metric with age and sex as covariates. As dynamic topological properties were obtained using the whole time-courses, not relying on any specific state, FDR correction was only used when comparing CV of nodal efficiency. The number of multiple comparison was 59—number of nodes, which was equal to the quantity of ICs used.

### Correlational analyses

Because the dynamic measures obtained in our study were non-normally distributed, we performed Spearman’s partial correlation analyses to investigate the possible relationships between abnormal properties and clinical data (including illness duration, VAS and attack frequency). Demographics (age, sex, education) and head motion (FD Jenkinson) were regressed out and *p <* 0.05 was set as the statistical significance threshold.

## Results

### Demographic and clinical characteristics

A total of 84 participants (41 CTN patients and 43 HCs) met the inclusion criteria and were included for analysis. Table 1 summarizes the detailed demographic and clinical data of the participants. There was no significant intergroup difference with respect to sex (*p* = 0.979), age (*p* = 0.186) and education (*p* = 0.412).

### Intrinsic connectivity networks

Based on the GICA framework, 59 independent components (ICs) were defined and selected, and their spatial maps of them are shown in Fig. 1. Specifically, all ICs were assigned into the following nine networks: sensorimotor network (SMN), visual network (VIS), auditory network (AUD), DMN, salience network (SN), cognitive control network (CC), dorsal attention network (DAN), subcortical network (SC), and cerebellum network (CBN). Figure 1B shows the static functional network connectivity (sFNC) matrix, computed with the entire BOLD time course and averaged over subjects. The detailed component labels and spatial information of ICs are presented in Supplementary Table S1 and Supplementary Fig. S2.

### Clustering analysis and functional connectivity strength in dynamic states

**Fig. 2:**
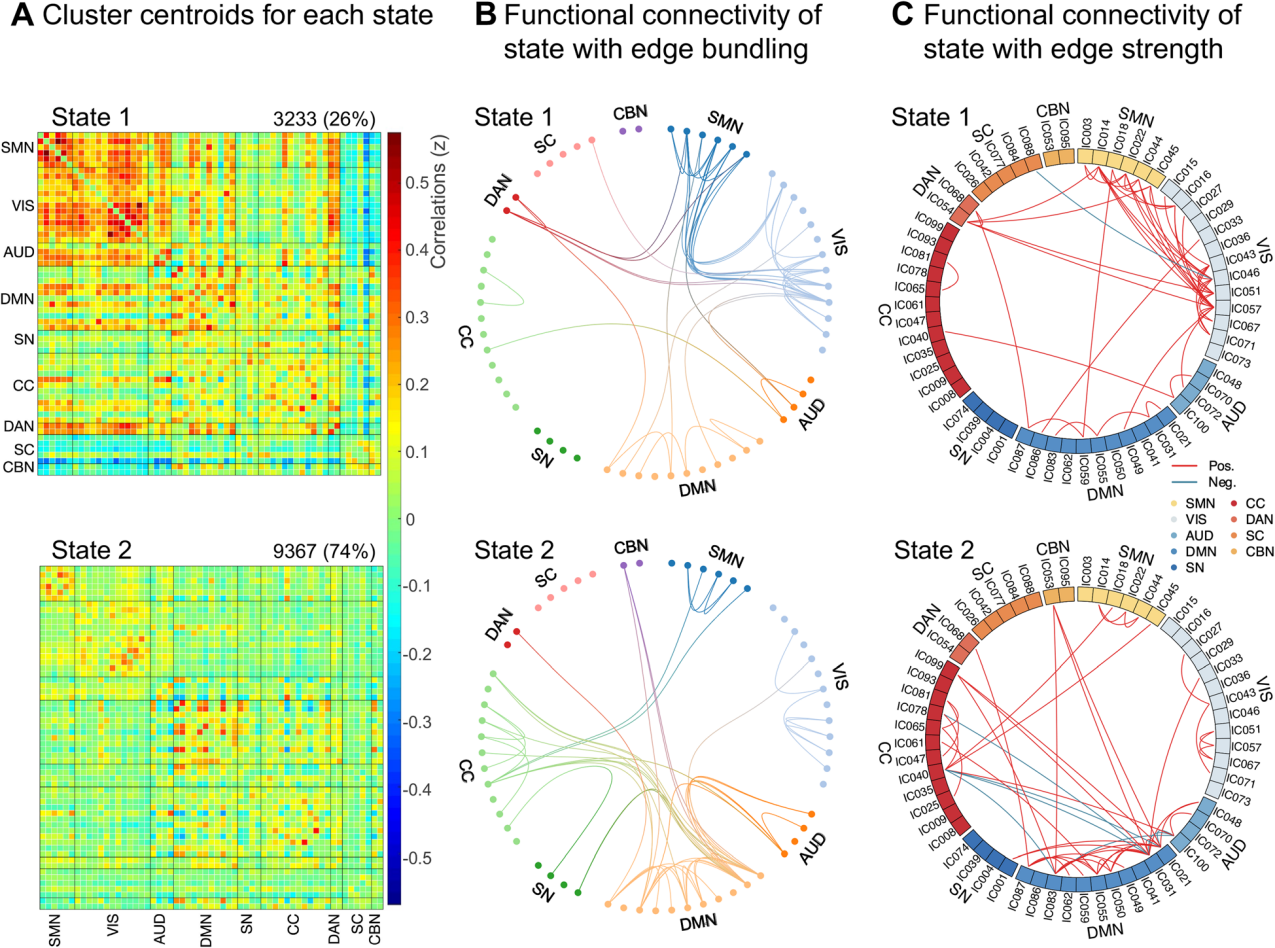
Clustering analysis results. (A) Cluster centroids for each state: state 1, less frequent but with stronger inter-connection; state 2, more frequent with relatively sparse connection. (B and C) The 3% strongest functional connections in each state are displayed, (absolute value of correlation coefficients was used), where B has edges bundled together for better characterizing connection patterns and C further shows connectivity type. The transition of colors in B means connections between networks. Red lines of C represent positive functional connectivity, while blue lines represent negative connections. SMN, sensorimotor network; VIS, visual network; AUD, auditory network; DMN, default mode network; SN, salience network; CC, cognitive control network; DAN, dorsal attention network; SC, subcortical network; CBN, cerebellar network

Through the evaluation of dynamic interactions between functional networks by sliding window and k-means clustering method, two recurred functional states of the whole cohort were identified as follows (Fig. 2A): a less frequent but strongly connected state 1 (26%) and a more frequent and sparsely connected state 2 (74%). For a more accurate description of connectivity patterns in each state, the 3% strongest functional connections are shown in Fig. 2B and C (with absolute strength of correlation coefficients as the index). State 1 was characterized by positive connections within and between SMN-VIS-DAN and widely negative connections between SC and other networks (though the absolute FC strength did not reach the top 3% except for IC088). State 2 was distinguished by partly strongly connected components within the DMN and complex coupling between DMN-CC (including both positive and negative correlations between ICs). Additionally, SN participated much more in state 2 than in state 1 and was highly connected with DMN.

**Fig. 3:**
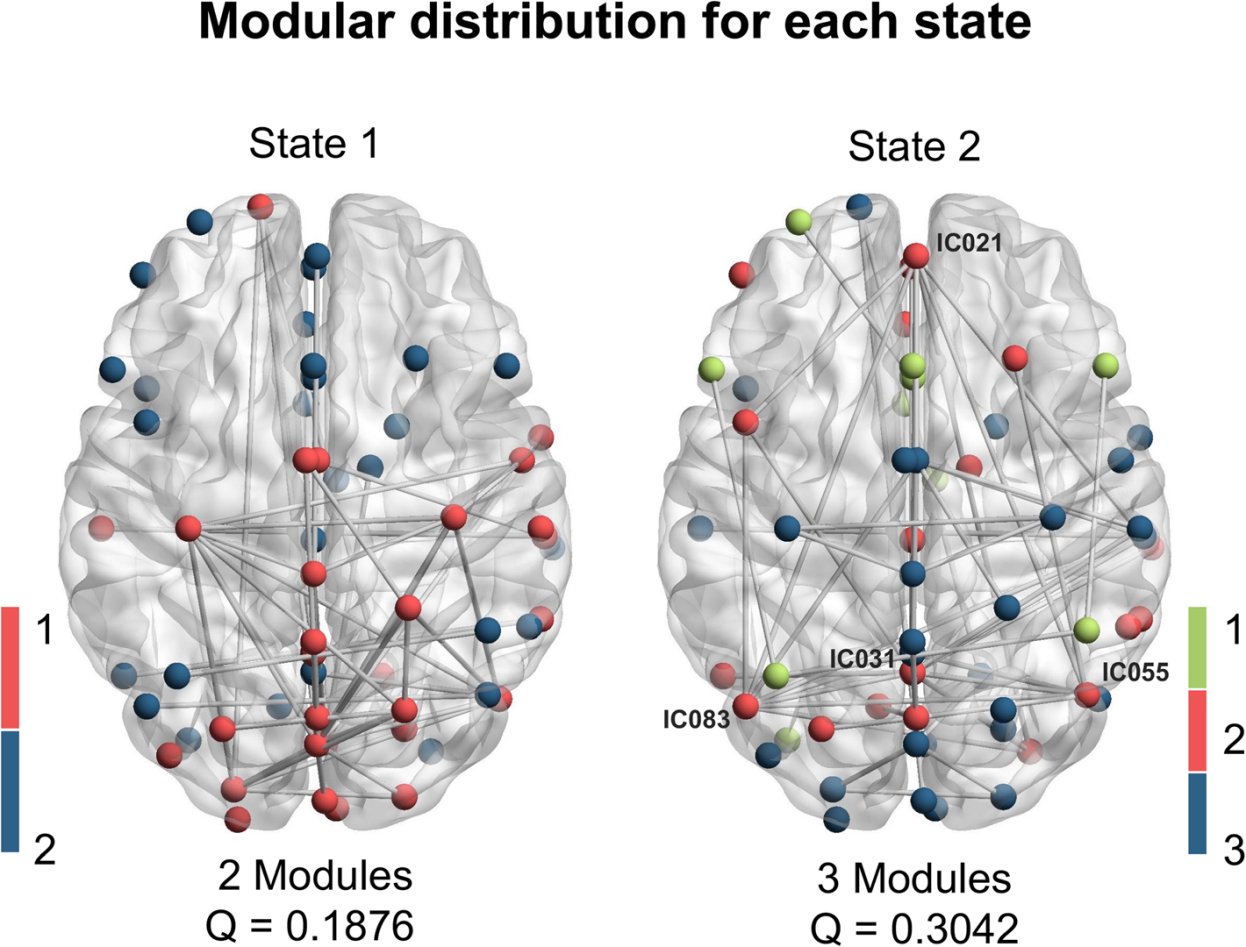
Modular analysis results. State 1 showed two modules (module 1 in red; module 2 in blue), whereas state 2 showed three modules (module 1 in green; module 2 in red, module 3 in blue). Edges between nodes represents 3% strongest functional connections in each state. In state 2, four nodes of module 2 are labeled with their components numbers because of their widespread connections, including IC21, IC31, IC55, IC83, all of which located in the DMN. IC, independent component. DMN, default mode network

The modularity analysis (Fig. 3) revealed quite distinct integration and segregation modes of the two states. With a lower Q (0.1876), state 1 presented two functional modules, one of which largely involved SMN, VIS, and DAN, while the other consisted of the other networks. By contrast, state 2 achieved a higher Q (0.3042), with ICs primarily aggregated into three modules. Among them, module 2 mainly included DMN and some part of CC and SN, that was predominant in the state 2 FC pattern described above.

### Group differences in temporal properties

**Fig. 4:**
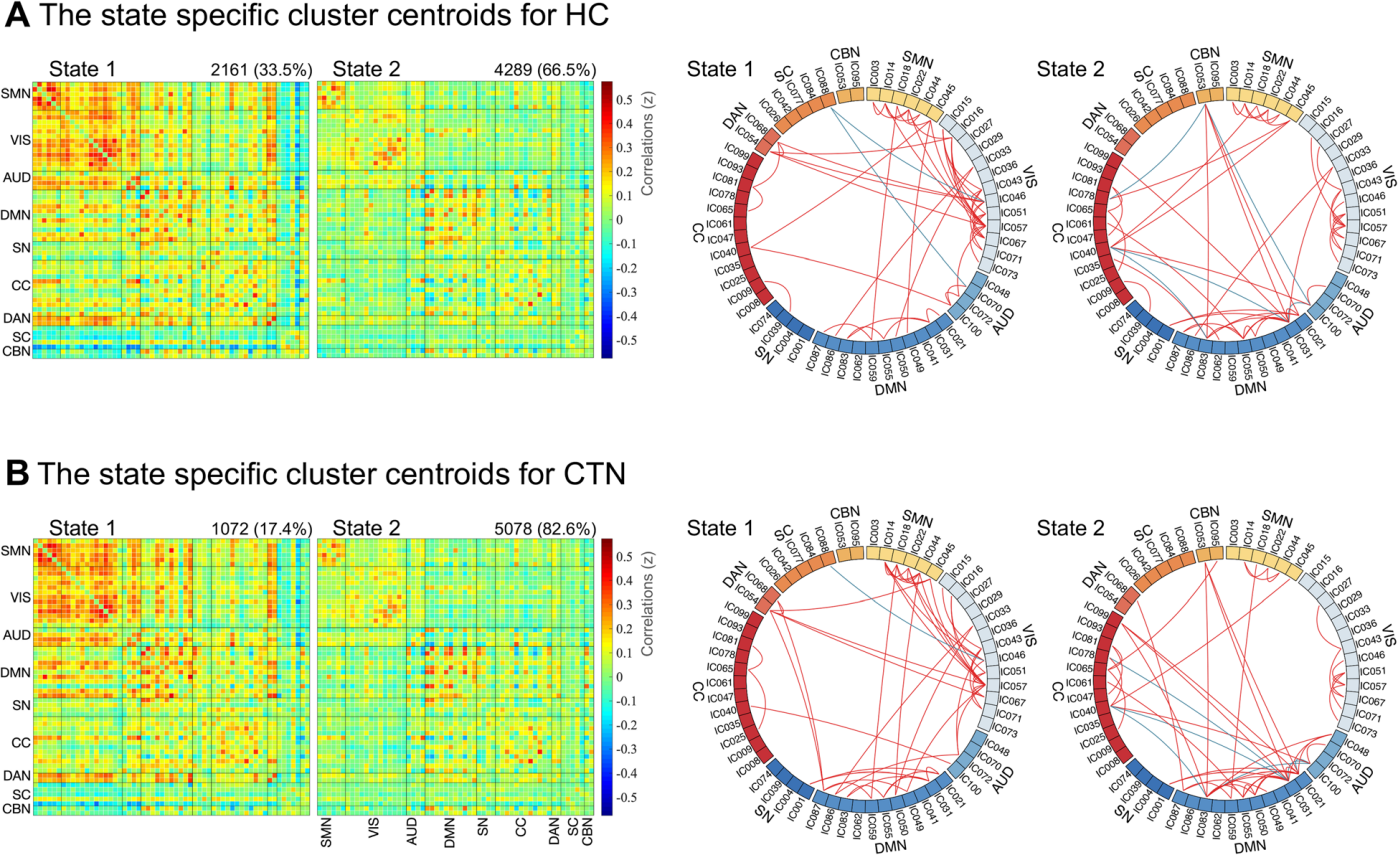
The two dFNC patterns of the two groups. (A) The state specific centroid matrices for HC. (B) The state specific centroid matrices for CTN. CTN, classic trigeminal neuralgia; HC, healthy controls; SMN, sensorimotor network; VIS, visual network; AUD, auditory network; DMN, default mode network; SN, salience network; CC, cognitive control network; DAN, dorsal attention network; SC, subcortical network; CBN, cerebellar network

**Fig. 5:**
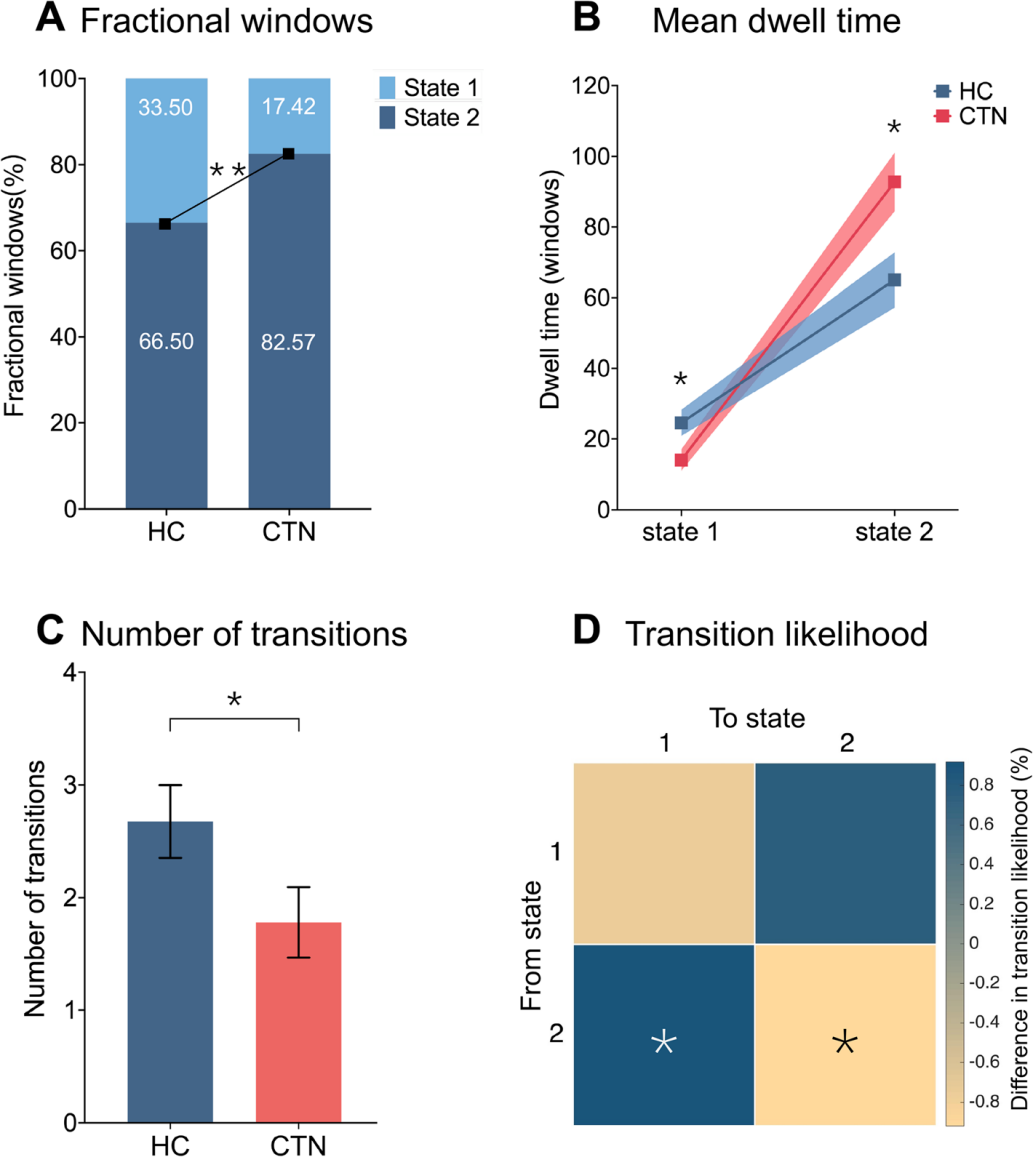
Analysis results of temporal properties. (A) Fractional windows, representing the percentage of all the windows in each state, (B) mean dwell time (i.e. the time duration a subject spent in each state) and (C) number of transitions (used to measure switching times between states) are displayed for CTN and HC. Square dots in B and bars in C reveal the mean values with shadow and error bar representing standard error. (D) Differences between groups in transition likelihood are shown. Asterisks (*) represent significance of *p <* 0.05 and asterisk (**) indicate *p <* 0.01 (FDR correction was used for fractional windows and mean dwell time, respectively). Taken together, CTN patients showed extreme preference for state 2, accompanied by decreased transition numbers and probabilities. CTN, classic trigeminal neuralgia; HC, healthy controls

The group-specific centroids of k-means clusters are shown in Fig. 4A and B. Although CTN and HC had similar dFNC profiles and connection patterns, we still found some significant group differences in the key temporal properties as shown in Fig. 5. In HC, the total occurrence of state 1 and state 2 was 33.5 ± 31.2% and 66.5 ± 31.2%, respectively. However, for CTN patients, a lower occurrence frequency was observed in state 1 (17.4 ± 25.5%), and a higher occurrence rate in state 2 (82.6 ± 25.5%), which differed significantly from HCs (*p =* 0.008, nonparametric permutation tests, FDR correction) (Fig. 5A). Accordingly, theses findings indirectly reflect that CTN patients had a decline in occurrence in state 1 by 16.1%, but a proportional rise was observed in state 2 (16.1%). Likewise, notable group discrepancy was identified for mean dwell time (*p =* 0.019, nonparametric permutation tests, FDR correction) (Fig. 5B). When compared to HC (mean ± SD for state 1: 24.6 ± 24.4; for state 2: 65.0 ± 51.1), CTN patients were inclined to spend a shorter time in state 1 (14.1 ± 19.3), but lingered for longer in state 2 (92.8 ± 53.1), suggesting an abnormal time distribution of patients for each state. Moreover, the transitions between the two states of CTN (2.7 ± 2.1) was significantly reduced when compared to HCs (1.8 ± 2.0) (*p =* 0.04, nonparametric permutation tests) (Fig. 5C).

When evaluating the transition likelihoods between two distinct states, substantial group differences regarding the probability of staying in a state and switching to another were found. Figure 5D shows that CTN patients preferred to stay in the sparsely connected state 2 (98.5 ± 2.8%, *p =* 0.026, nonparametric permutation tests), and were less likely to switch to the strongly connected state 1 (1.5 ± 2.8%, *p =* 0.026, nonparametric permutation tests), which is entirely opposite in HC (mean ± SD for staying in state 2: 96.9 ± 4.5%; for transition to state 1: 3.1 ± 4.5%). However, there was no group difference with respect to the preference of staying in state 1 (mean ± SD for CTN: 3.3 ± 4.1%; for HC: 96.7 ± 4.1%, *p =* 0.374, nonparametric permutation tests) or transferring to state 2 (mean ± SD for CTN: 3.0 ± 3.6%; for HC: 97.0 ± 3.6%, *p =* 0.378, nonparametric permutation tests). The results are consistent with and further support the findings about fractional window and dwell time. In summary, these results indicated an affection to the stability of strong connections in state 1 in CTN patients, with a proportionate increase in expression of sparse connections in state 2. Correlation analysis did not find any relationships between temporal metrics and clinical characteristics.

In validation analysis, when the window size was set to 16-TR and 24-TR with the rest of parameters unchanged, two cluster states were obtained of each run. State 1 and state 2 under both window sizes (16-TR and 24-TR) showed a similar FC pattern to the ones under 20-TR window size (refer to supplementary Table S4 and S5 for detailed *r* and *p*). We also observed consistent between-groups differences in temporal metrics under both window sizes (Fig. S3 and S4).

### Dynamic topological properties

**Fig. 6:**
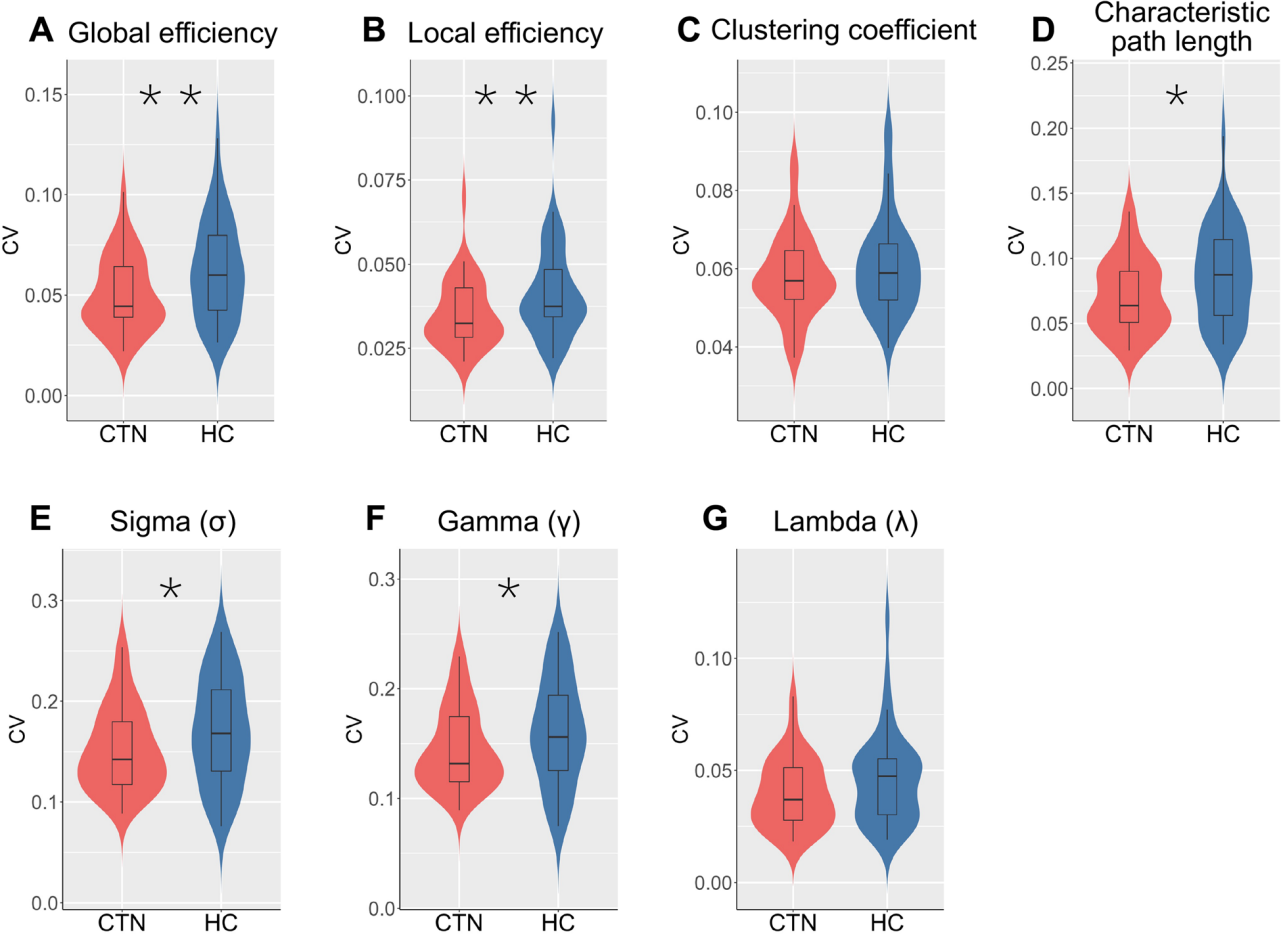
CV comparing of global topological properties (AUC). Violin plots (A ~ G) represent CV of AUC of global efficiency, local efficiency, clustering coefficient, characteristic path length, sigma, gamma, and lambda, respectively for CTN (red) and HC (blue). All asterisks indicate a significant group differences (*, *p <* 0.05; **, *p <* 0.01). Horizontal lines in boxes indicate group medians. CV, coefficient of variation; CTN, classic trigeminal neuralgia; HC, healthy controls

**Fig. 7:**
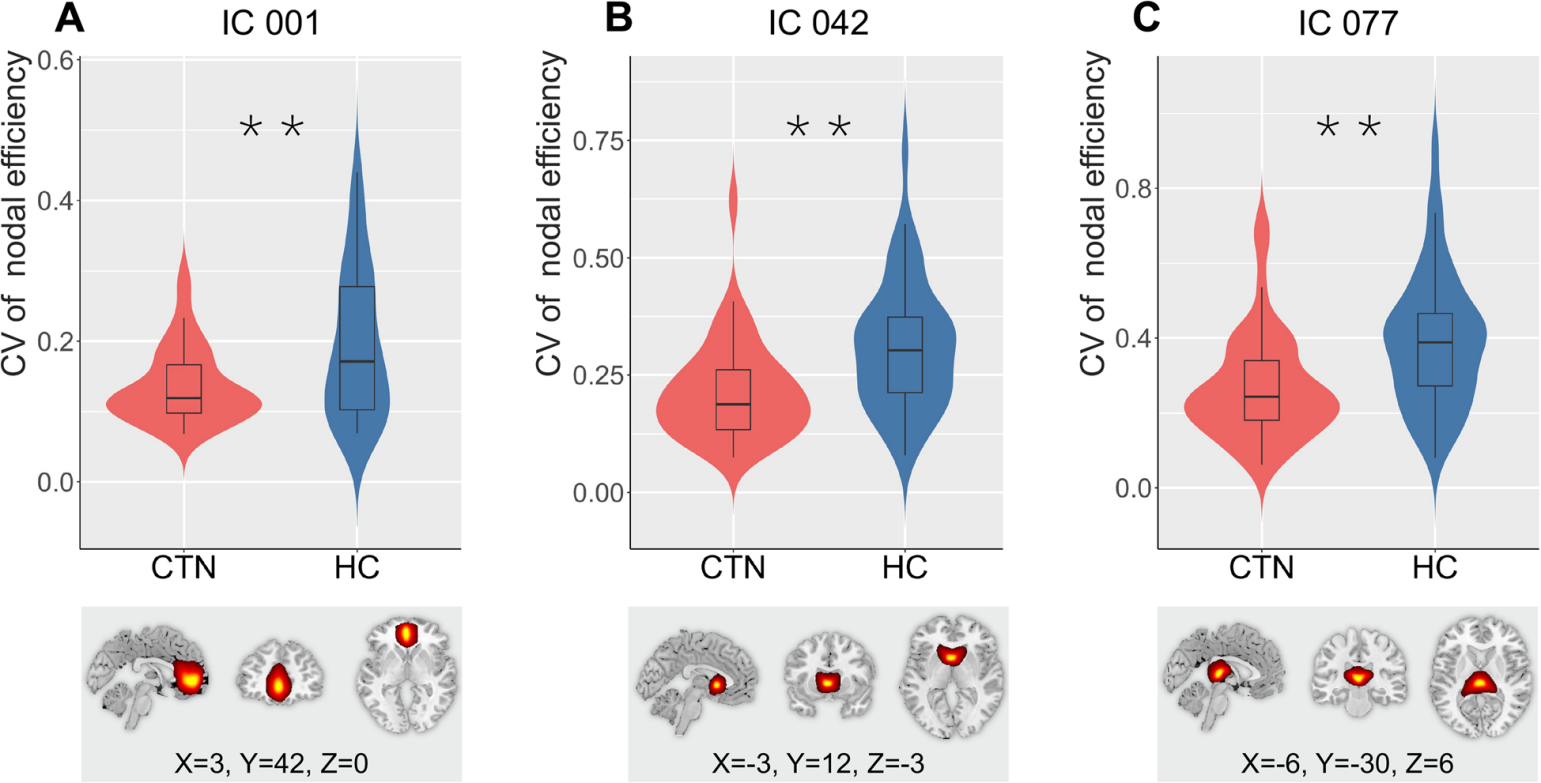
CV comparing of nodal efficiency (AUC). The CV for AUC of nodal efficiency of (A) IC1 (located in ACC), (B) IC42 (located in caudate) and (C) IC77 (located in thalamus) are displayed using violin plots for CTN (red) and HC (blue). All asterisks indicate a significant group difference (**, *p <* 0.01, FDR corrected). Horizontal lines in boxes indicate group medians. CV, coefficient of variation; CTN, classic trigeminal neuralgia; HC, healthy controls; IC, independent component; ACC, anterior cingulate cortex

Significant differences between CTN and HC were identified when comparing CV for AUC of network efficiency (*p =* 0.007 for E_g_ and *p =* 0.06 for E_loc_, nonparametric permutation tests) (Fig. 6A and B) and small-world metrics (*p =* 0.029 for *σ*; *p =* 0.035 for *γ*; *p =* 0.017 for *L*, nonparametric permutation tests). Still, we did not find abnormal alterations of patients in dynamics of AUC of *λ* and *C* (*p =* 0.074 and *p =* 0.138 respectively, nonparametric permutation tests) (Fig. 6C–G). With regard to the temporal variability of nodal efficiency (AUC), CTN patients showed decreased values in IC 1, IC 42 and IC 77 (*p =* 0.001 for IC 1 and IC 77; *p =* 0.002 for IC 42, FDR corrected). IC 1 (peak MNI coordinate: 3, 42, 0) is mainly located in the anterior cingulate cortex (ACC) (Fig. 7A); IC 42 (peak MNI coordinate: − 3, 12, − 3) is mainly located in the bilateral caudates nucleus and IC 77 (peak MNI coordinate: − 6, − 30, 6) is mainly located in bilateral thalamus, both of which belong to the SC network.

**Fig. 8:**
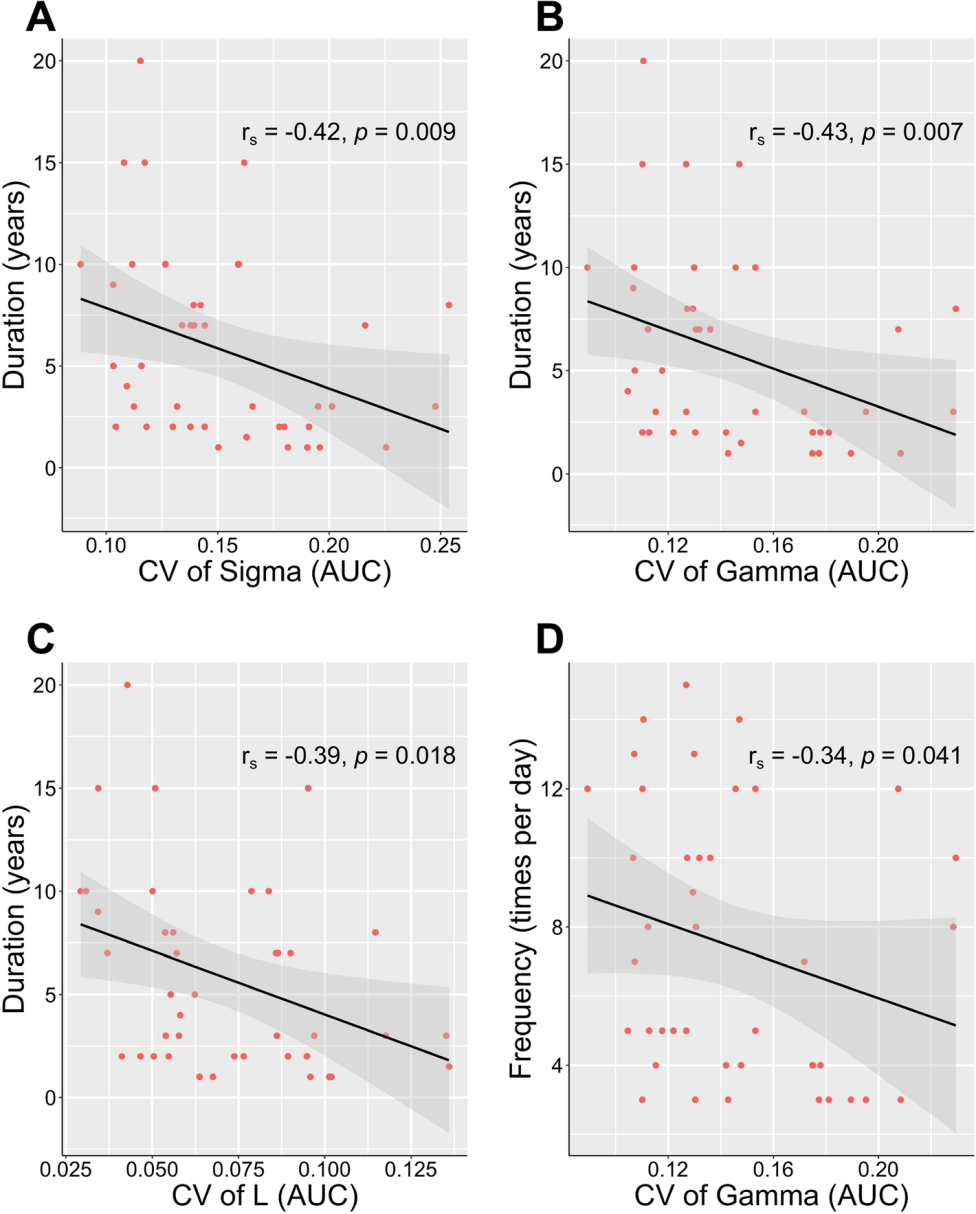
Correlation between clinical characteristics and CV of topological properties (AUC) for CTN group. (A ~ C) The disease duration was negatively correlated with CV of AUC of Sigma, Gamma, and characteristic path length. (D) The CV of Gamma showed negative correlation with attack frequency. *L*, characteristic path length; CV, coefficient of variation; CTN, classic trigeminal neuralgia

In the further analysis of correlations between dynamic topological properties and clinical data in the CTN group, we found that the CV of AUC of *σ*, *γ,* and *L* were negatively correlated with disease duration (Spearman’s rho = − 0.421, − 0.433, and − 0.388 respectively; and uncorrected *p =* 0.009, 0.018, and 0.007, respectively) (Fig. 8A–C). Additionally, the CV of gamma (AUC) was negatively correlated with the pain attack frequency (Spearman’s rho = − 0.338, uncorrected *p =* 0.041) (Fig. 8D).

## Discussion

Investigation about temporal features of FC has been proven valuable to reflect neural mechanisms of pain development [50]. To our best knowledge, this is the first dFNC study combined with graph theory to investigate the temporal properties of states and variability of the whole brain topological organization in CTN patients. Based on the two reoccurring dFNC states with distinct connectivity configuration: an infrequent state 1 with strong connections and a frequent state 2 with sparse connections, there were three major findings associated with CTN: (I) Patients showed more fractional windows and longer dwell time in state 2 than state 1, which is predominantly characterized by tight connections between DMN and CC and locally positive connections within DMN; (II) CTN patients demonstrated decreased transition numbers, paralleled by disruptions of variability in both global (including network efficiency and small-worldness) and local (nodal efficiency) topological properties, which suggested an impaired flexibility of information transfer in patients. More importantly, the damages in dynamics of nodal efficiency highlighted the crucial role of the ACC, thalamus and caudate nucleus in the pathophysiology of CTN; (III) The negative correlation between global dynamic properties and disease duration as well as attack frequency further suggested a clinical relevance.

### dFNC states

#### Increased reoccurrence fraction in state 2

In the present study, we found that CTN patients spent more time in state 2, characterized by extensively sparse connections but with strong FC within DMN and between DMN and CC. In previous studies, such weak and diffuse dFNC state, like state 2 we observed, was always considered steadier and as the average of a vast number of additional states that varying less [14, 51], which may also explain the similarity between state 2 and sFNC pattern. Some studies further linked the state with self-referential processing and drowsiness [52, 53]. One previous study about DMN activity in temporomandibular disorder (TMD) suggested that pain rumination in patients were positively correlated to FC within DMN [54]. Also, by using arterial spin labeling, TN patients and TMD patients both displayed increased cerebral blood flow in dorsal precuneus [55]. Hence, the prolonged dwell time of patients in such static state may reflect pain rumination, or persistent negative thinking about pain. Correspondingly, in state 2 we found some connections between DMN and DAN. With similar dFNC analysis, Tu et al. found that patients with migraine had reduced expression of DMN, which was proposed to stem from weaker alpha band oscillation and eventually lead to reduced mind-wandering experience [23]. Conversely, our findings are consistent with an electroencephalography study about chronic orofacial neuropathic pain, which demonstrated significantly greater activity over the theta and alpha ranges in patients [56] that reversely supported the increased DMN activation. Altogether, give the CTN is characterized by long-term paroxysmal nociceptive input, patients may assign more attention to pain [57], further manifest as enhanced communication within the DMN.

Tight and complicated FC between DMN and CC also characterized state 2, including extensive positive connections and several negative connections. As a “task-positive” network, CC engages in external stimuli and tasks, and would be activated significantly in attention and executive control to modulate the descending pain system under pain-related stimuli. Thus, CC generally exhibits negative FC with DMN [58, 59]. One recent rs-fMRI study on chronic migraine revealed disrupted negative FC between the DMN and CC of patients [58]. Another study investigated the migraine brain using dynamic amplitude of low-frequency fluctuations (dALFF) and found decreased dynamics in both DMN and CC [60]. In the present study, the higher occurrence of a state with obvious FC between DMN and CC may indicate the state is a neural substrate for the dysregulation of static FC between networks in CTN. The disturbance of DMN-CC decoupling possibly reflects an imbalance of switching between internally and externally directed cognition and further influence cognitive and emotional processing of pain [61].

Additionally, the ICs in state 2 were subdivided into three modules with higher modularity index, interpreted as stronger segregation between neural network groups [62]. As indicated in previous studies, dysfunction in integration characterizes pain such as migraine, CTN, and other neuropathic pain [63, 64], and may facilitate the processing of pain-related information [65]. Furthermore, the disconnections between modules probably reflect interruptions on inter-system communication [65] and impairments in cognitive performance that are known to be a complication of pain [16, 66]. Therefore, the increased fractional and mean dwell time, and highest modularity in state 2 in patients indicates increased periods of excessive functional segregation in CTN and potentially a potentially reduced ability to flexibly switch to state 1.

#### Decreased reoccurrence fraction in state 1

In our study, state 1 was characterized by widely positive connections, especially within and between SMN-VIS-DAN, all of which are parts of the sensory system and participate in the information processing of external stimuli [61]. With regard to the SMN, an increasing number of studies have demonstrated the central role of the somatosensory cortex in processing and modulating pain [12].

The somatic motor cortex has been linked to pain processing by providing feedback from various layers to the distinct thalamic nucleus anatomically [67]. It has been reported that CTN patients commonly show mild hypoesthesia [2, 68]. Previous morphological research about CTN showed decreased gray matter volume (GMV) in the SMN, including secondary somatosensory cortex, primary motor cortex, and premotor area [9]. Thus, our findings provided further functional evidence to support the injury to SMN. In addition, pain experience is accompanied by several sensory inputs, such as visual, auditory, and olfactory, which may interfere with each other. Altered FC within VIS and AUD networks has been revealed in previous functional research about chronic migraine [61]. Taken together, the observed decreased expression of sensory network-related FC may suggest the failed modulation between and within sensory-related networks, probably leading to attenuated perception in CTN patients.

We also observed some obvious negative connections between SC and cortical networks in state 1. We speculate that the shorter duration of patients in the strongly connected state may partly reflect the dysfunction of cortical-subcortical interaction, which was confirmed by the following analysis (see “Variability of nodal efficiency analysis” for details). Other dFNC studies of multiple diseases including low back pain [24], migraine [23], schizophrenia [69], and bipolar disorder [70] also showed similar state with aberrant FC between SC and disease-related cortical areas, wherein all diseases share a common thalamocortical dysrhythmia model. Thus, the present findings may provide supportive evidence of dFNC analysis to declare the neural mechanism by separating temporally contiguous states alone.

### Temporal variability of topological metrics

Consistent with the decreased number of transitions, CTN patients displayed robustly reduced temporal variability of global topological properties. As the integration and segregation of large-scale brain network serially fluctuates time [14], dynamic topological analysis can provide additional information. In our study, CV represents the discreteness of network windows and further reflects the flexibility of rapid shift between mental states, which may be beneficial for maintaining the responsive ability of the brain [71], as well as help optimize behavior during pain for a better task performance [72]. Furthermore, reduced dynamics in E_g_, E_loc_, *σ*, *γ* and *L* of CTN patients probably suggested an impairment on flexibility of the global functional networks. Similarly, Wu et al found altered temporal stability of global parameters of primary dysmenorrhea [25], which suggested dynamically reorganization of brain network. Moreover, the CV of topological properties in our study was found to be correlated with disease duration and attack frequency. As reported in previous studies, the efficiency of neural systems is partly reflected by the temporal variability of neural activity [73]. It has been proven that altered temporal variability was not only related to lower thermal pain threshold but also could be used as predictor of pain characteristics [73, 74]. Thus, our findings may imply less efficient information transfer throughout the whole brain in patients. The variability of topological properties may be supposed as potential indices in CTN management.

Regarding to the nodal efficiency, regions with decreased temporal variability were observed mainly located in the ACC of SN as well as the thalamus and caudate nucleus of SC. As the key nodes of SN, ACC plays a critical role in marking salient events (such as pain) for further processing and providing controls for better cognitive and behavioral response [75]. Accordingly, the function of SN seems to be entirely contrary to DMN—activated when attention was focused on pain, and suppressed otherwise [16]. Previous studies have found increased FC between insular and ACC [9] in CTN patients, along with decreased GMV [76, 77]. Moreover, ACC is involved in the rewarding effects of pain relief and displays tight coupling with brainstem pain-control circuit (such as periaqueductal gray [PAG] and locus ceruleus [LC]) to provide regulation from the high neural system [78]. Thus, in the present study, reduced the dynamics of ACC efficiency in CTN may induce abnormal modulation and switching between SN and other “dynamic pain connectome” regions, thereby leading to dysfunction in coping with changing environments and needs.

The thalamus is a part of the ascending pain pathway [6], and functions as a relay and integration center connecting subcortical and cortical regions. Previous investigations using electroencephalography and magnetoencephalography techniques have shown that chronic neuropathic pain is associated with thalamocortical dysrhythmia [79, 80]. Likewise, rs-fMRI study demonstrated increased infra-slow oscillation activity of the thalamus [6]. In our dFNC study, we further suggested reduced temporal variability in the functional efficiency of the thalamus, especially located in the ventroposterior medial region. In general, the ventroposterior medial thalamus is under inhibition of γ-aminobutyric acid (GABA) released from the thalamic reticular nucleus; this process was supposed to play a key role in controlling thalamocortical rhythm [81, 82]. Moreover, aberrant thalamic firing, especially increased burst firing in the somatosensory thalamus without overall hyperactivity, has been proven to be associated with neuropathic pain [69]. With respect to our results in temporal fluctuation, it has been indicated that signal variability was associated with the balance of synaptic excitation and inhibition, where the greater variability may represent better neuronal plasticity, for further adaptation to the changing environment [83, 84]. Therefore, attenuated flexibility of thalamic efficiency in CTN may be linked to excessive thalamic firing, either as a predisposing factor or consequence, subsequently perhaps to contribute to the vulnerability of thalamocortical connectivity and cause constant perception of pain.

The caudate nucleus is another key nucleus of the SC network and receive nociceptive information from the trigeminal nuclei through direct projections from lamina I neurons of the trigeminal spinal nucleus, and independently of the thalamus [85]. The caudate nucleus plays a critical role in the evaluation of the agreement between the action and the outcome, as well as planning and performing tasks necessary to achieve complex goals [86]. Several morphological studies have suggested reduced GMV of the caudate nucleus in chronic pain disorders such as TN [77], cluster headache [87], and knee osteoarthritis [86]. Consistent with those studies, our findings provided additional functional evidence from the perspective of dynamic topology. Thus, the lower efficiency of the caudate nucleus may be partly explained by an adaptation to chronic stimulation or an inhibition of facial movement to avoid eliciting pain [3, 9].

### Limitation

Our study has some limitations. Firstly, the patients enrolled were all taking medications, most commonly carbamazepine. Thus, drug effects should be considered and controlled in the future study to diminish the influences [88]. Moreover, given the attack side was not uniform in our patients, though no statistically significant difference was found in subgroup analysis (see supplementary material), it is needed to validate with large samples. Additionally, multiband acquisition allows scanning with a shorter TR and elevated temporal resolution [89]. In future investigations, it would be expected to increase the estimation power by using fast fMRI. Finally, multimodal approaches including GM and WM morphological analysis as well as GABA related metabolic research need to be used on more brain regions, such as the PAG and the rostroventral medulla from the antinociceptive system. By applying machine learning and related methods, it is expected to identify more fine-grained changes in “dynamic pain connectome” and valuable indices.

## Conclusion

To our knowledge, this is the first study to assess dynamic connectivity properties of CTN. Abnormal temporal patterns, characterized by complex connections between DMN-CC and hyper-connectivity within DMN, were found mainly in patients. Additionally, we observed disrupted flexibility in state transition and global topological organization that furthermore identified key brain regions (ACC in the SN and the thalamus and caudate nucleus in the SC) with decreased temporal variability of efficiency. Reduced dynamics of topological properties were further correlated with both disease duration and pain frequency. These results collectively suggested a temporal disturbance of whole brain networks due to chronic pain and further highlighted the crucial role of “dynamic pain connectome” regions (including DMN/CC/SN) in the pathophysiology of CTN, also provided supplementary evidence for current knowledge about the dysfunction of cortical-subcortical interaction in pain development.

## Supplementary Information

**Additional file 1.**

## Data Availability

All data generated and analyzed during the current study will be available from the corresponding author on reasonable request.

## Abbreviations

ACC: Anterior cingulate cortex
AUD: Auditory network
CBN: Cerebellar network
CC: Cognitive control network
CTN: Classic trigeminal neuralgia
dFNC: Dynamic functional network connectivity
DAN: Dorsal attention network
DMN: Default mode network
HC: Healthy controls
ICs: Independent components
SC: Subcortical network
sFC: Static functional connectivity
SMN: Sensorimotor network
SN: Salience network
VAS: Visual analogue scale
VIS: Visual network

## References

[1] Scholz J, Finnerup NB, Attal N (2019). The IASP classification of chronic pain for ICD-11: chronic neuropathic pain. Pain.

[2] Headache Classification Committee (2018). Headache classification Committee of the International Headache Society (IHS) the international classification of headache disorders, 3rd edition. Cephalalgia.

[3] Cruccu G, Di Stefano G, Truini A (2020). Trigeminal Neuralgia. N Engl J Med.

[4] Obermann M, Yoon MS, Ese D (2007). Impaired trigeminal nociceptive processing in patients with trigeminal neuralgia. Neurology.

[5] Leonard G, Goffaux P, Mathieu D (2009). Evidence of descending inhibition deficits in atypical but not classical trigeminal neuralgia. Pain.

[6] Alshelh Z, Di Pietro F, Youssef AM (2016). Chronic neuropathic pain: It's about the rhythm. J Neurosci.

[7] Tian T, Guo L, Xu J (2016). Brain white matter plasticity and functional reorganization underlying the central pathogenesis of trigeminal neuralgia. Sci Rep.

[8] Tsai YH, Liang X, Yang JT (2019). Modular organization of brain resting state networks in patients with classical trigeminal neuralgia. Neuroimage Clin.

[9] Wang Y, Cao DY, Remeniuk B (2017). Altered brain structure and function associated with sensory and affective components of classic trigeminal neuralgia. Pain.

[10] Wang Y, Zhang Y, Zhang J (2018). Structural and functional abnormalities of the insular cortex in trigeminal neuralgia: a multimodal magnetic resonance imaging analysis. Pain.

[11] Zhang Y, Mao Z, Pan L (2019). Frequency-specific alterations in cortical rhythms and functional connectivity in trigeminal neuralgia. Brain Imaging Behav.

[12] Henssen D, Dijk J, Knepfle R (2019). Alterations in grey matter density and functional connectivity in trigeminal neuropathic pain and trigeminal neuralgia: a systematic review and meta-analysis. Neuroimage Clin.

[13] Hutchison RM, Womelsdorf T, Allen EA (2013). Dynamic functional connectivity: promise, issues, and interpretations. Neuroimage.

[14] Allen EA, Damaraju E, Plis SM (2014). Tracking whole-brain connectivity dynamics in the resting state. Cereb Cortex.

[15] Chang C, Glover GH (2010). Time-frequency dynamics of resting-state brain connectivity measured with fMRI. Neuroimage.

[16] Kucyi A, Davis KD (2015). The dynamic pain connectome. Trends Neurosci.

[17] Rahman QA, Janmohamed T, Pirbaglou M (2018). Defining and predicting pain volatility in users of the manage my pain app: analysis using data mining and machine learning methods. J Med Internet Res.

[18] Bushnell MC, Ceko M, Low LA (2013). Cognitive and emotional control of pain and its disruption in chronic pain. Nat Rev Neurosci.

[19] Wiech K, Tracey I (2009). The influence of negative emotions on pain: behavioral effects and neural mechanisms. Neuroimage.

[20] Necka EA, Lee IS, Kucyi A (2019). Applications of dynamic functional connectivity to pain and its modulation. Pain Rep.

[21] Yan J, Li M, Fu S (2019). Alterations of dynamic regional homogeneity in trigeminal neuralgia: a resting-state fMRI study. Front Neurol.

[22] Lee MJ, Park BY, Cho S (2019). Dynamic functional connectivity of the migraine brain: a resting-state functional magnetic resonance imaging study. Pain.

[23] Tu Y, Fu Z, Zeng F (2019). Abnormal thalamocortical network dynamics in migraine. Neurology.

[24] Tu Y, Fu Z, Mao C (2020). Distinct thalamocortical network dynamics are associated with the pathophysiology of chronic low back pain. Nat Commun.

[25] Wu X, Yu W, Hu H et al (2021) Dynamic network topological properties for classifying primary dysmenorrhoea in the pain-free phase. Eur J Pain. 10.1002/ejp.1808 10.1002/ejp.1808 34008281

[26] De Lacy N, Doherty D, King BH (2017). Disruption to control network function correlates with altered dynamic connectivity in the wider autism spectrum. Neuroimage Clin.

[27] Zhu PW, Chen Y, Gong YX (2020). Altered brain network centrality in patients with trigeminal neuralgia: a resting-state fMRI study. Acta Radiol.

[28] Luo L, Li Q, You W (2021). Altered brain functional network dynamics in obsessive-compulsive disorder. Hum Brain Mapp.

[29] Chao-Gan Y, Yu-Feng Z (2010). DPARSF: a MATLAB toolbox for “pipeline” data analysis of resting-state fMRI. Front Syst Neurosci.

[30] Calhoun VD, Adali T, Pearlson GD (2001). A method for making group inferences from functional MRI data using independent component analysis. Hum Brain Mapp.

[31] Roweis S (1997). EM Algorithms for PCA and SPCA. advances in neural information processing systems.

[32] Bell AJ, Sejnowski TJ (1995). An information-maximization approach to blind separation and blind deconvolution. Neural Comput.

[33] Himberg J, Hyvarinen A, Esposito F (2004). Validating the independent components of neuroimaging time series via clustering and visualization. Neuroimage.

[34] Calhoun VD, Adali T, Pearlson GD (2001). Spatial and temporal independent component analysis of functional MRI data containing a pair of task-related waveforms. Hum Brain Mapp.

[35] Ma S, Correa NM, Li XL (2011). Automatic identification of functional clusters in FMRI data using spatial dependence. IEEE Trans Biomed Eng.

[36] Allen EA, Erhardt EB, Damaraju E (2011). A baseline for the multivariate comparison of resting-state networks. Front Syst Neurosci.

[37] Cordes D, Haughton VM, Arfanakis K (2000). Mapping functionally related regions of brain with functional connectivity MR imaging. AJNR Am J Neuroradiol.

[38] Fiorenzato E, Strafella AP, Kim J (2019). Dynamic functional connectivity changes associated with dementia in Parkinson's disease. Brain.

[39] Kim J, Criaud M, Cho SS (2017). Abnormal intrinsic brain functional network dynamics in Parkinson's disease. Brain.

[40] Weng Y, Liu X, Hu H (2020). Open eyes and closed eyes elicit different temporal properties of brain functional networks. Neuroimage.

[41] Preti MG, Bolton TA, Van De Ville D (2017). The dynamic functional connectome: state-of-the-art and perspectives. Neuroimage.

[42] Varoquaux G, Gramfort A, Poline JB et al (2010) Brain covariance selection: better individual functional connectivity models using population prior. arXiv. preprint arXiv:10085071

[43] Friedman J, Hastie T, Tibshirani R (2008). Sparse inverse covariance estimation with the graphical lasso. Biostatistics.

[44] Aggarwal CC, Hinneburg A, Keim DA (2000). On the surprising behavior of distance metrics in high dimensional spaces. Paper presented at the Database Theory - Icdt.

[45] Rousseeuw PJ (1987). Silhouettes - a graphical aid to the interpretation and validation of cluster-analysis. J Comput Appl Math.

[46] Yue Q, Martin RC, Fischer-Baum S (2017). Brain modularity mediates the relation between task complexity and performance. J Cogn Neurosci.

[47] Wu X, He H, Shi L (2019). Personality traits are related with dynamic functional connectivity in major depression disorder: a resting-state analysis. J Affect Disord.

[48] Zheng W, Zhao Z, Zhang Z (2021). Developmental pattern of the cortical topology in high-functioning individuals with autism spectrum disorder. Hum Brain Mapp.

[49] Nour MM, Dahoun T, Mccutcheon RA et al (2019) Task-induced functional brain connectivity mediates the relationship between striatal D2/3 receptors and working memory. Elife 8. 10.7554/eLife.45045 10.7554/eLife.45045 PMC6620042 31290741

[50] Mutso AA, Petre B, Huang L (2014). Reorganization of hippocampal functional connectivity with transition to chronic back pain. J Neurophysiol.

[51] Fu Z, Caprihan A, Chen J (2019). Altered static and dynamic functional network connectivity in Alzheimer's disease and subcortical ischemic vascular disease: shared and specific brain connectivity abnormalities. Hum Brain Mapp.

[52] Marusak HA, Calhoun VD, Brown S (2017). Dynamic functional connectivity of neurocognitive networks in children. Hum Brain Mapp.

[53] Allen EA, Damaraju E, Eichele T (2018). EEG signatures of dynamic functional network connectivity states. Brain Topogr.

[54] Kucyi A, Moayedi M, Weissman-Fogel I (2014). Enhanced medial prefrontal-default mode network functional connectivity in chronic pain and its association with pain rumination. J Neurosci.

[55] Youssef AM, Gustin SM, Nash PG (2014). Differential brain activity in subjects with painful trigeminal neuropathy and painful temporomandibular disorder. Pain.

[56] Di Pietro F, Macey PM, Rae CD (2018). The relationship between thalamic GABA content and resting cortical rhythm in neuropathic pain. Hum Brain Mapp.

[57] Tu Y, Jung M, Gollub RL (2019). Abnormal medial prefrontal cortex functional connectivity and its association with clinical symptoms in chronic low back pain. Pain.

[58] Coppola G, Di Renzo A, Petolicchio B (2019). Aberrant interactions of cortical networks in chronic migraine: a resting-state fMRI study. Neurology.

[59] Davey CG, Pujol J, Harrison BJ (2016). Mapping the self in the brain's default mode network. Neuroimage.

[60] Chen H, Qi G, Zhang Y (2021). Altered dynamic amplitude of Low-frequency fluctuations in patients with migraine without Aura. Front Hum Neurosci.

[61] Zou Y, Tang W, Qiao X (2021). Aberrant modulations of static functional connectivity and dynamic functional network connectivity in chronic migraine. Quant Imaging Med Surg.

[62] Newman ME, Girvan M (2004). Finding and evaluating community structure in networks. Phys Rev E Stat Nonlinear Soft Matter Phys.

[63] Schwedt TJ, Schlaggar BL, Mar S (2013). Atypical resting-state functional connectivity of affective pain regions in chronic migraine. Headache.

[64] Michels L, Koirala N, Groppa S (2021). Structural brain network characteristics in patients with episodic and chronic migraine. J Headache Pain.

[65] Zheng W, Woo CW, Yao Z (2020). Pain-evoked reorganization in functional brain networks. Cereb Cortex.

[66] Buhle J, Wager TD (2010). Does meditation training lead to enduring changes in the anticipation and experience of pain?. Pain.

[67] Sherman SM (2016). Thalamus plays a central role in ongoing cortical functioning. Nat Neurosci.

[68] Bendtsen L, Zakrzewska JM, Heinskou TB (2020). Advances in diagnosis, classification, pathophysiology, and management of trigeminal neuralgia. Lancet Neurol.

[69] Lenz FA, Kwan HC, Dostrovsky JO (1989). Characteristics of the bursting pattern of action potentials that occurs in the thalamus of patients with central pain. Brain Res.

[70] Demirtas M, Tornador C, Falcon C (2016). Dynamic functional connectivity reveals altered variability in functional connectivity among patients with major depressive disorder. Hum Brain Mapp.

[71] Zalesky A, Fornito A, Cocchi L (2014). Time-resolved resting-state brain networks. Proc Natl Acad Sci U S A.

[72] Cheng JC, Bosma RL, Hemington KS (2017). Slow-5 dynamic functional connectivity reflects the capacity to sustain cognitive performance during pain. Neuroimage.

[73] Lim M, Jassar H, Kim DJ (2021). Differential alteration of fMRI signal variability in the ascending trigeminal somatosensory and pain modulatory pathways in migraine. J Headache Pain.

[74] Rogachov A, Cheng JC, Hemington KS (2018). Abnormal Low-frequency oscillations reflect trait-like pain ratings in chronic pain patients revealed through a machine learning approach. J Neurosci.

[75] Menon V, Uddin LQ (2010). Saliency, switching, attention and control: a network model of insula function. Brain Struct Funct.

[76] Obermann M, Rodriguez-Raecke R, Naegel S (2013). Gray matter volume reduction reflects chronic pain in trigeminal neuralgia. Neuroimage.

[77] Li M, Yan J, Li S (2017). Reduced volume of gray matter in patients with trigeminal neuralgia. Brain Imaging Behav.

[78] Mills EP, Di Pietro F, Alshelh Z (2018). Brainstem pain-control circuitry connectivity in chronic neuropathic pain. J Neurosci.

[79] Sarnthein J, Stern J, Aufenberg C (2006). Increased EEG power and slowed dominant frequency in patients with neurogenic pain. Brain.

[80] Stern J, Jeanmonod D, Sarnthein J (2006). Persistent EEG overactivation in the cortical pain matrix of neurogenic pain patients. Neuroimage.

[81] Fuentealba P, Steriade M (2005). The reticular nucleus revisited: intrinsic and network properties of a thalamic pacemaker. Prog Neurobiol.

[82] Zhang M, Liu S, Wang S (2021). Reduced thalamic resting-state functional connectivity and impaired cognition in acute abstinent heroin users. Hum Brain Mapp.

[83] Garrett DD, Samanez-Larkin GR, Macdonald SW (2013). Moment-to-moment brain signal variability: a next frontier in human brain mapping?. Neurosci Biobehav Rev.

[84] Shew WL, Yang H, Petermann T (2009). Neuronal avalanches imply maximum dynamic range in cortical networks at criticality. J Neurosci.

[85] Chudler EH, Dong WK (1995). The role of the basal ganglia in nociception and pain. Pain.

[86] Mao CP, Bai ZL, Zhang XN (2016). Abnormal subcortical brain morphology in patients with knee osteoarthritis: a cross-sectional study. Front Aging Neurosci.

[87] Absinta M, Rocca MA, Colombo B (2012). Selective decreased grey matter volume of the pain-matrix network in cluster headache. Cephalalgia.

[88] Liu S, Wang S, Zhang M (2021). Brain responses to drug cues predict craving changes in abstinent heroin users: a preliminary study. Neuroimage.

[89] Bhandari R, Kirilina E, Caan M (2020). Does higher sampling rate (multiband + SENSE) improve group statistics - an example from social neuroscience block design at 3T. Neuroimage.

