# Supplementary material for: Altered brain functional network dynamics in classic trigeminal neuralgia: a resting-state functional magnetic resonance imaging study
Source: J Headache Pain. 2021 Dec 11;22(1):147. doi: 10.1186/s10194-021-01354-z (PMC8903588; doi:10.1186/s10194-021-01354-z)
Supplement: Supplementary file 1 — Additional file 1. [file 10194_2021_1354_MOESM1_ESM.pdf]

Supplementary Information

Altered brain functional network dynamics in classic trigeminal neuralgia: a resting-state functional magnetic resonance imaging study

Pengfei Zhang[1, 2#], Yanli Jiang[1, 2#], Guangyao Liu[1, 2], Jiao Han[1], Jun Wang[1, 2], Laiyang Ma[1, 2], Wanjun Hu[2], Jing Zhang[2*]

[*]Address correspondence to:

Prof. **Jing Zhang**, Department of Magnetic Resonance, Lanzhou University Second Hospital, Cuiyingmen No.82, Chengguan District, Lanzhou, 730030, PR China.

E-mail: **lztong2001@163.com**

I. Supplementary Methods

II. Supplementary Tables (Table S1～S6)

III. Supplementary Figures (Figure S1～S17)

I. Supplementary Methods

Modularity analysis

Modularity analysis was performed with the Brain Connectivity Toolbox (www.brain-connectivity-toolbox.net/). Specifically, Louvain algorithm was used to define group level modularity and then Q was obtained to reflect network segregation and describe the degree to which a network may be subdivided into non-overlapping communities. Given the stochastic nature of the Louvain algorithm, consensus clustering approach was applied to ensure the robustness of the final community structure. For each subject we iteratively run the consensus algorithm 1000 times to yield a single agreement matrix (D), where D_{ij} represents the proportion of partitions in which nodes *i* and *j* were assigned to the same community. If the proportion was lower than 50%, the agreement value would be 0. Subsequently, Louvain algorithm run for another 1000 times on the consensus matrix to obtain one consensus modular partition. Next, processes mentioned above repeated until there was a single consensus partition[1,2].

K-means clustering analysis

In our study, k-means clustering was done with "temporal dFNC" toolbox in GIFT. Clustering algorithm was applied on 150 window FC matrices for all subjects, with L1 distance (Manhattan distance) to estimate the similarity between matrix and cluster centroids. To avoid the bias of initial random selection of cluster centroids, 100 iteration of k-means algorithm was used on subsampling windows, which were selected with composed of local maxima in functional connectivity variance for lower

redundancy between windows as well as computational demand. Further, as a well-known method, Silhouette method has been suggested preferable for estimating the potential optimal cluster number in most cases for many distinct scenarios[3]. As in previous studies[4,5], a cluster validity analysis (Silhouette) was carried out on the exemplars of all the subjects to estimate the optimal number of clusters.

II. Supplementary Tables

Table S1. Peak activation of 59 independent components in the present study.

Intrinsic connectivity network		*Nv*	*T*-value	MNI coordinate[§]		
				x	y	z
**Sensorimotor network (SMN)**						
IC003	Postcentral_R	1426	24.35	57	-6	24
	Postcentral_L	1124	21.12	-48	-15	36
IC014	Precentral_L	2626	20.77	-36	-30	60
IC018	Supp_Motor_Area_R	1414	19.42	6	-9	69
IC022	Postcentral_R	2097	22.77	48	-24	48
	Postcentral_L	209	8.37	-48	-27	51
IC044	Paracentral_Lobule_R	1848	27.66	3	-36	63
IC045	Postcentral_R	1301	21.97	24	-48	63
	Parietal_Sup_L	1039	20.92	-27	-45	63
**Visual network (VIS)**						
IC015	Lingual_R	1249	26.02	9	-90	-3
IC016	Temporal_Inf_R	1379	22.53	51	-63	-3
	Occipital_Mid_L	874	17.90	-48	-69	0
IC027	Calcarine_L	1173	27.83	-3	-93	12
IC029	Occipital_Mid_L	915	25.39	-21	-99	-3
	Occipital_Inf_R	750	23.49	24	-96	-6
IC033	Temporal_Mid_L	2682	25.83	-54	-60	18
	Frontal_Inf_Orb_L	377	13.77	-48	24	-12
IC036	Occipital_Mid_L	1030	23.50	-42	-78	18
	Occipital_Mid_R	1113	19.52	42	-75	21
IC043	Calcarine_L	2159	25.94	-12	-66	3
IC046	Calcarine_R	1644	27.62	6	-72	6
IC051	Occipital_Mid_L	2061	27.99	-30	-90	9
	Occipital_Mid_R	342	8.85	30	-87	6
IC057	Cuneus_L	1761	31.74	-3	-75	30
IC067	Occipital_Sup_R	1593	25.88	18	-93	15
	Occipital_Mid_L	235	9.22	-24	-90	18
IC071	Calcarine_R	1989	25.15	15	-66	12
IC073	Fusiform_R	1446	23.38	24	-75	-15
	Fusiform_L	475	10.29	-24	-78	-9
**Auditory network (AUD)**						
IC048	Temporal_Mid_R	1620	23.52	57	-30	-6
	Temporal_Mid_L	201	7.49	-60	-27	0
IC070	Temporal_Sup_L	2856	19.89	-54	-24	9

IC072	Temporal_Sup_R	1188	22.00	60	-6	3
	Temporal_Sup_L	886	15.76	-57	-12	3
IC100	Temporal_Sup_R	1483	30.73	57	-24	15
	Temporal_Sup_L	569	15.31	-54	-27	12
**Default mode network (DMN)**						
IC021	Frontal_Sup_Medial_L	2419	25.12	-3	48	39
IC031	Precuneus_L	1717	32.12	0	-66	30
IC041	Cingulum_Mid_L	2629	22.72	0	-27	36
IC049	Precuneus_L	2485	31.09	-6	-54	48
IC050	Precuneus_L	1591	32.17	-3	-57	9
IC055	Angular_R	1058	28.70	51	-63	27
	Angular_L	561	18.05	-42	-72	39
	Cingulum_Mid_R	201	9.30	3	-39	39
	Frontal_Mid_R	425	8.14	30	24	42
IC059	Precuneus_R	1108	26.01	3	-48	69
IC062	Cuneus_L	2552	31.35	-9	-69	30
IC083	Angular_L	1346	30.04	-42	-66	42
	Frontal_Mid_L	1185	11.95	-36	21	45
	Cingulum_Mid_L	338	9.81	-3	-36	33
IC086	Temporal_Mid_R	1503	25.58	54	-51	9
	Temporal_Mid_L	272	8.81	-54	-51	9
IC087	Precuneus_R	1266	28.51	3	-66	54
**Salience network (SN)**						
IC001	Cingulum_Ant_R	2482	27.22	3	42	0
IC004	Rectus_L	1655	25.96	-3	36	-15
IC039	Cingulum_Mid_L	2724	25.57	0	18	36
IC074	Frontal_Inf_Orb_L	1126	27.29	-45	18	-9
	Frontal_Inf_Orb_R	1016	26.72	39	21	-12
**Cognitive control network (CC)**						
IC008	Supp_Motor_Area_L	1395	27.77	-3	18	57
IC009	Frontal_Mid_L	2702	21.12	-27	57	6
IC025	Frontal_Inf_Tri_R	2413	25.98	48	21	21
	Frontal_Inf_Tri_L	423	10.70	-51	18	21
IC035	Frontal_Mid_Orb_L	1108	19.80	-33	57	-6
	Frontal_Sup_Orb_R	1333	16.44	30	57	-6
IC040	Cingulum_Mid_L	3328	23.34	0	-3	48
	Temporal_Pole_Sup_R	316	10.42	57	6	0
IC047	Frontal_Inf_Tri_L	3239	23.10	-54	21	6
	Frontal_Inf_Tri_R	285	8.68	48	39	9
	Parietal_Inf_L	305	7.83	-51	-42	42
IC061	Frontal_Sup_R	3457	21.68	24	18	48

IC065	SupraMarginal_R	1583	26.55	51	-42	42
	Temporal_Inf_R	289	11.71	57	-48	-15
	Frontal_Mid_R	279	8.37	42	45	6
IC078	Parietal_Inf_R	1643	20.29	48	-33	51
	SupraMarginal_L	1207	19.37	-60	-30	36
IC081	Parietal_Sup_L	2017	28.43	-30	-60	54
IC093	SupraMarginal_R	1399	30.77	57	-45	27
	SupraMarginal_L	640	18.09	-57	-54	30
	Temporal_Mid_R	255	13.39	57	-21	-12
IC099	Precentral_L	1212	15.90	-42	6	48
	Precentral_R	1118	11.00	33	-3	45
	Parietal_Inf_R	393	10.35	30	-54	54
**Dorsal attention network (DAN)**						
IC054	Angular_R	1699	29.13	27	-66	48
	Parietal_Sup_L	451	18.06	-21	-69	48
IC068	Occipital_Mid_L	1621	31.04	-27	-75	39
**Subcortical network (SC)**						
IC026	Putamen_R	1545	19.96	21	9	-6
	Putamen_L	1254	17.18	-21	6	-6
IC042	Caudate_L	1233	28.77	-3	12	-3
IC077	Thalamus_L	1302	25.85	-6	-30	6
IC084	Thalamus_R	1490	26.45	0	-15	12
IC088	Caudate_L	3593	13.72	-15	-9	24
**Cerebellum network (CB)**						
IC053	Cerebelum_Crus1	2182	25.22	30	-81	-33
IC095	Cerebelum_Crus1	1369	19.61	-33	-78	-33
	Angular_L	378	8.96	-36	-66	42
	Frontal_Mid_R	259	7.86	36	60	6

Note: *§* The coordinates are peak voxel coordinates of the one-sample t-test results for each independent component spatial maps of all subjects with the threshold of $t > \mu + 4\sigma$, k > 200. IC, independent component; Nv, number of voxel in each cluster; R, right; L, left; M, medial.

Table S2. Mathematical definitions and interpretations of topological network measures used in the present study.

Topological parameters	Definitions	Descriptions
Global network efficiency (E_g)	$E_g(G) = \frac{1}{N(N-1)} \sum_{i=1}^{N} \sum_{j=1, i \neq j}^{N} \frac{1}{L_{ij}}$	E_g measures the efficiency of parallel information transfer over the whole network. L_{ij} is the shortest path length between node *i* and node *j*[6].
Local network efficiency (E_{loc})	$E_{loc}(G) = \frac{1}{N} \sum_{i=1}^{N} E_g(G_i)$	E_{loc} represents the fault tolerance of the network, reflecting the capability of information exchange for each subgraph when the index node is eliminated[7].
Smallworldness (σ)	$\sigma = \gamma/\lambda$	When the balance between the segregation and integration of information processing is well obtained, network will show smallworldness and σ will be larger than 1, wherein most nodes in network not adjacent with each other directly but with efficient information transfer[8].
Normalized clustering coefficient (γ)	$\gamma = C/C_{rand}$	A network with smallworldness has a higher clustering coefficient than a random network, resulting in $\gamma = C/C_{rand} > 1$, where The C_{rand} represents the clustering coefficient of random networks[8].
Normalized characteristic path length (λ)	$\lambda = L/L_{rand}$	The characteristic path length of a small-world network is similar to random networks, thus$\lambda = L/L_{rand} \approx 1$, where the L_{rand} represents the characteristic path length of random networks[8].
Clustering coefficient (C)	$C(G) = \frac{2}{k_i(k_i - 1)} \sum_{j,k} (\overline{w}_{ij}\overline{w}_{jk}\overline{w}_{ki})^{1/3}$	C describes the level of local neighborhood clustering within a graph, expressing the extent of local cliquishness in a network. k_i represents the degree of node *i*[8].
Characteristic path length (L)	$L(G) = \frac{1}{N(N-1)} \sum_{i=1}^{N} \sum_{j=1, i \neq j}^{N} L_{ij}$	L_p provides information about close on average a node of the network is connected to every other node in the network, reflecting how efficient information can be integrated over the whole brain network[9].
Nodal efficiency	$E_{nodal} = \sum_{m \neq i \neq n \in G} \frac{\sigma_{mn}(i)}{\sigma_{mn}}$	Nodal reflects the efficiency of information propagation from one node to the rest nodes of network[7].

Table S3. Temporal properties of dynamic functional network connectivity (dFNC) states with different window length in CTN and HC.

Window length	Temporal properties	dFNC States	CTN	HC	*p value*
16-TR	fractional windows	hyper-connected	17.4±24.1%	31.3±29.2%	0.010
		hypo-connected	82.6±24.1%	68.7±29.2%	0.010
	mean dwell time	hyper-connected	12.5± 19.0	20.4±20.3	0.036
		hypo-connected	85.1±54.6	58.9±50.5	0.024
	number of transitions	—	2.5±2.4	3.5±2.6	0.039
24-TR	fractional windows	hyper-connected	17.2±26.0%	35.3±32.9%	0.004
		hypo-connected	82.8±26.0%	64.7±32.9%	0.004
	mean dwell time	hyper-connected	14.5± 20.1	28.1±30.6	0.009
		hypo-connected	95.0±50.9	66.9±52.1	0.009
	number of transitions	—	2.3±2.1	1.6±2.1	0.084

Note: Based on sliding window approach, we validated our results at different window sizes (32 s and 48 s). Two dFNC states were obtained by using the *k*-means clustering algorithm and silhouette criterion. State 1 was referred to hyper-connected state because of tightly positive connections within and between networks. Conversely, state 2 was referred to hypo-connected state because of extensively sparse connections. Values were displayed as mean±SD (range). *p* value was calculated by nonparametric permutation tests (10,000 repetition) and FDR correction was used for fractional windows and mean dwell time.

Table S4. The correspondence between pairs of centroids in two dynamic functional network connectivity (dFNC) states under the two different window sizes (20-TR and 16-TR).

**Window size = 16-TR**	**Window size = 20-TR**	
	state 1	state 2
state 1	r = 0.999* (p<0.001)	r = 0.723 (p<0.001)
state 2	r = 0.732 (p<0.001)	r = 0.999* (p<0.001)

Note: r represents Pearson's correlation coefficient and * indicates the high similarity of state centroids under the two different window sizes.

Table S5. The correspondence between pairs of centroids in two dynamic functional network connectivity (dFNC) states under the two different window sizes (20-TR and 24-TR).

**Window size = 24-TR**	**Window size = 20-TR**	
	state 1	state 2
state 1	r = 0.999* (p<0.001)	r = 0.742 (p<0.001)
state 2	r = 0.734 (p<0.001)	r = 0.999* (p<0.001)

Note: r represents Pearson's correlation coefficient and * indicates the high similarity of state centroids under the two different window sizes.

Table S6. Comparisons between CTN patients suffering from different side of pain.

**Properties**	**dFNC States**	**CTN_R**	**CTN_L**	***p value***
Sex (female/male)	—	16/8	7/10	0.105
Age, y	—	59.83±9.00	51.41±10.73	0.010*
Education, y	—	12.04±2.24	11.12±2.42	0.215
Duration of	—	5.65±4.28	6.00±5.33	0.815

disease, y				
Attack frequency (times per day)	—	7.42±3.96	7.41±3.96	0.997
Score of VAS	—	6.60±0.98	6.14±0.75	0.111
fractional windows	state 1	11.9±19.4%	25.2±29.9%	0.2588
	state 2	88.1±19.4%	74.8±29.9%	0.2588
mean dwell time	state 1	9.57±11.7	20.4±24.8	0.4925
	state 2	100.12±51.3	82.4±52.3	0.4925
number of transitions	—	1.54±1.71	2.12±2.27	0.4474
CV of Eg (AUC)	—	0.048±0.016	0.054±0.020	0.4437
CV of Eloc (AUC)	—	0.033±0.007	0.039±0.012	0.1118
CV of σ (AUC)	—	0.143±0.032	0.165±0.47	0.2430
CV of γ (AUC)	—	0.137±0.027	0.157±0.043	0.2077
CV of λ (AUC)	—	0.041±0.016	0.038±0.014	0.0855
CV of C (AUC)	—	0.058±0.010	0.057±0.012	0.3792
CV of L (AUC)	—	0.071±0.029	0.073±0.025	0.2439

Note: Results of subgroup analysis. Values were displayed as mean±SD (range). *p* value of sex was calculated by chi-square test and *p* values of age, education, and VAS were obtained by independent-samples t-test. Differences of temporal properties and dynamic topological properties were obtained by 10,000 nonparametric permutation tests, where sex and age were treated as covariates. As *p* values of fractional windows and mean dwell time were acquired in each state, FDR method was used for correction. Statistically significant difference between groups was only found in age (*p* value was labeled *). CTN, classic trigeminal neuralgia; AUC, area under curve; R, right; L, left.

Table S8. Differences of temporal properties and dynamic topological metrics in the FSL validation analysis.

Properties	dFNC States	CTN	HC	*p value*
fractional windows	hyper-connected	21.9±24.6%	39.3±32.7%	0.0044**
	hypo-connected	78.1±24.6%	60.7±32.7%	0.0044**
mean dwell time	hyper-connected	15.6±18.4	24.7±23.8	0.028*
	hypo-connected	76.1±51.0	53.8±50.3	0.028*
number of transitions	—	2.63±2.36	3.37±2.39	0.093

Note: Values were displayed as mean±SD (range). *p* values of all temporal properties were obtained by 10,000 nonparametric permutation tests with sex and age treated as covariates. As *p* values of fractional windows and mean dwell time were acquired in each state, FDR method was used for correction. * represents $p<0.05$, and ** represents $p<0.01$. CTN, classic trigeminal neuralgia; HC, health controls.

Table S9. Differences of temporal properties and dynamic topological metrics in the validation analysis with Power atlas.

Properties	dFNC States	CTN	HC	*p value*
fractional windows	hyper-connected	17.5±23.4%	30.8±27.2%	0.0128*
	hypo-connected	82.5±23.4%	69.2±27.2%	0.0128*
mean dwell time	hyper-connected	9.88±11.22	15.81±15.68	0.0338*
	hypo-connected	82.23±54.74	55.25±51.24	0.028*
number of transitions	—	3.00±3.04	4.72±3.57	0.01*

Note: Values were displayed as mean±SD (range). *p* values of all temporal properties were obtained by 10,000 nonparametric permutation tests with sex and age treated as covariates. As *p* values of fractional windows and mean dwell time were acquired in

each state, FDR method was used for correction. * represents $p<0.05$. CTN, classic trigeminal neuralgia; HC, health controls.

Table S10. Differences of temporal properties and dynamic topological metrics in the validation analysis with Yeo atlas.

Properties	dFNC States	CTN	HC	*p value*
fractional windows	hyper-connected	17.48±26.16%	34.43±31.02%	0.0048**
	hypo-connected	82.52±26.16%	65.57±31.02%	0.0048**
mean dwell time	hyper-connected	13.79±20.19	25.78±24.70	0.0076**
	hypo-connected	94.36±53.79	62.99±49.01	0.0076**
number of transitions	—	1.78±1.94	2.65±2.02	0.0319*
CV of Eg (AUC)	—	0.044±0.016	0.054±0.023	0.0121*
CV of Eloc (AUC)	—	0.031±0.009	0.037±0.013	0.0055**
CV of σ (AUC)	—	0.143±0.044	0.162±0.055	0.0384*
CV of γ (AUC)	—	0.137±0.038	0.153±0.049	0.0357*
CV of λ (AUC)	—	0.034±0.013	0.039±0.017	0.0643
CV of C (AUC)	—	0.055±0.012	0.057±0.012	0.2051
CV of L (AUC)	—	0.061±0.025	0.076±0.035	0.0148*

Note: Values were displayed as mean±SD (range). *p* values of all temporal properties were obtained by 10,000 nonparametric permutation tests with sex and age treated as covariates. As *p* value of fractional windows and mean dwell time were acquired in each state, FDR method was used for correction. * represents $p<0.05$, and ** represents $p<0.01$. CTN, classic trigeminal neuralgia; HC, health controls.

III. Supplementary Figures

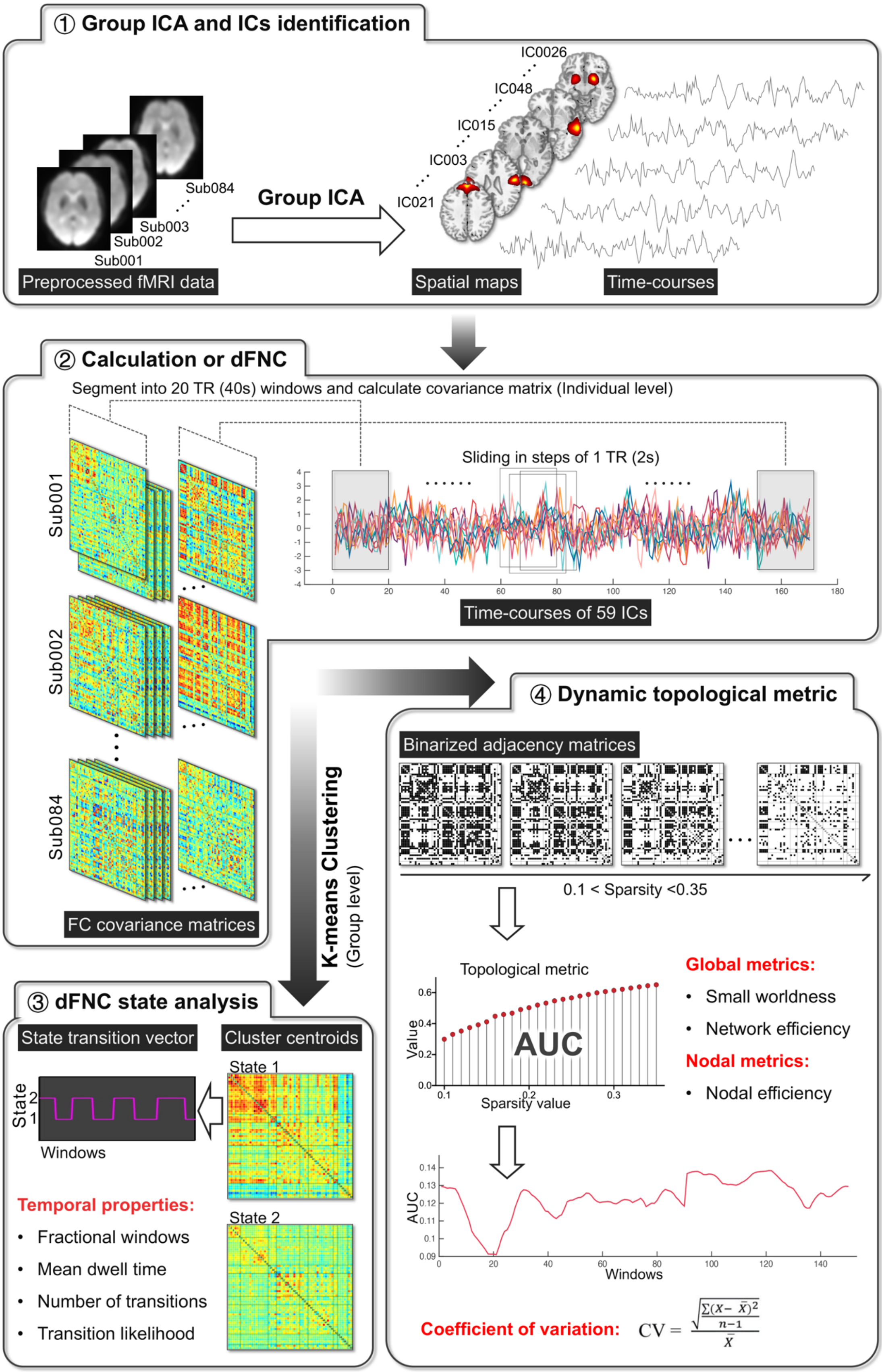

$$\mathrm{CV} = \frac{\sqrt{\frac{\sum (X - \bar{X})^2}{n-1}}}{\bar{X}}$$

Figure S1. Flowchart of dynamic functional network connectivity (dFNC) state analysis and dynamic topological analysis. ① Group independent component analysis (GICA) was used to decompose preprocessed fMRI date into 100 independent components (ICs). Then, 59 ICs were selected and assigned into nine networks; ② Based on sliding-window approach, time-coursed of each individual were segmented into 150 windows (window size was 20 TR, 40s) with steps of 1 TR (2s). In every window, the FC covariance matrices of the 59 ICs were calculated; ③ FC matrices of all subjects were clustered using k-means algorithm and two dFNC states were obtained. Four temporal properties, including fractional windows, mean dwell time, number of transitions and transition likelihood, were calculated for all participants; ④ In dynamic graph theoretical analysis, global and nodal topological properties were computed across all windows and all subjects under a series of sparsity thresholds (from 0.10 to 0.35, with an interval of 0.01). The coefficient of variation (CV) of the area under the curve (AUC) of metrics were calculated to represent temporal variability.

The optimal number of k by using Silhouette

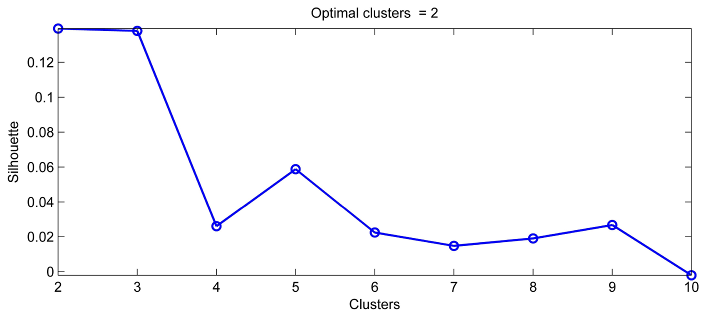

Figure S2. The optimal number of k. The silhouette criterion was used to estimate the potential optimal cluster number. It is when the silhouette coefficient is close to 1 that indicates the sample is better clustered.

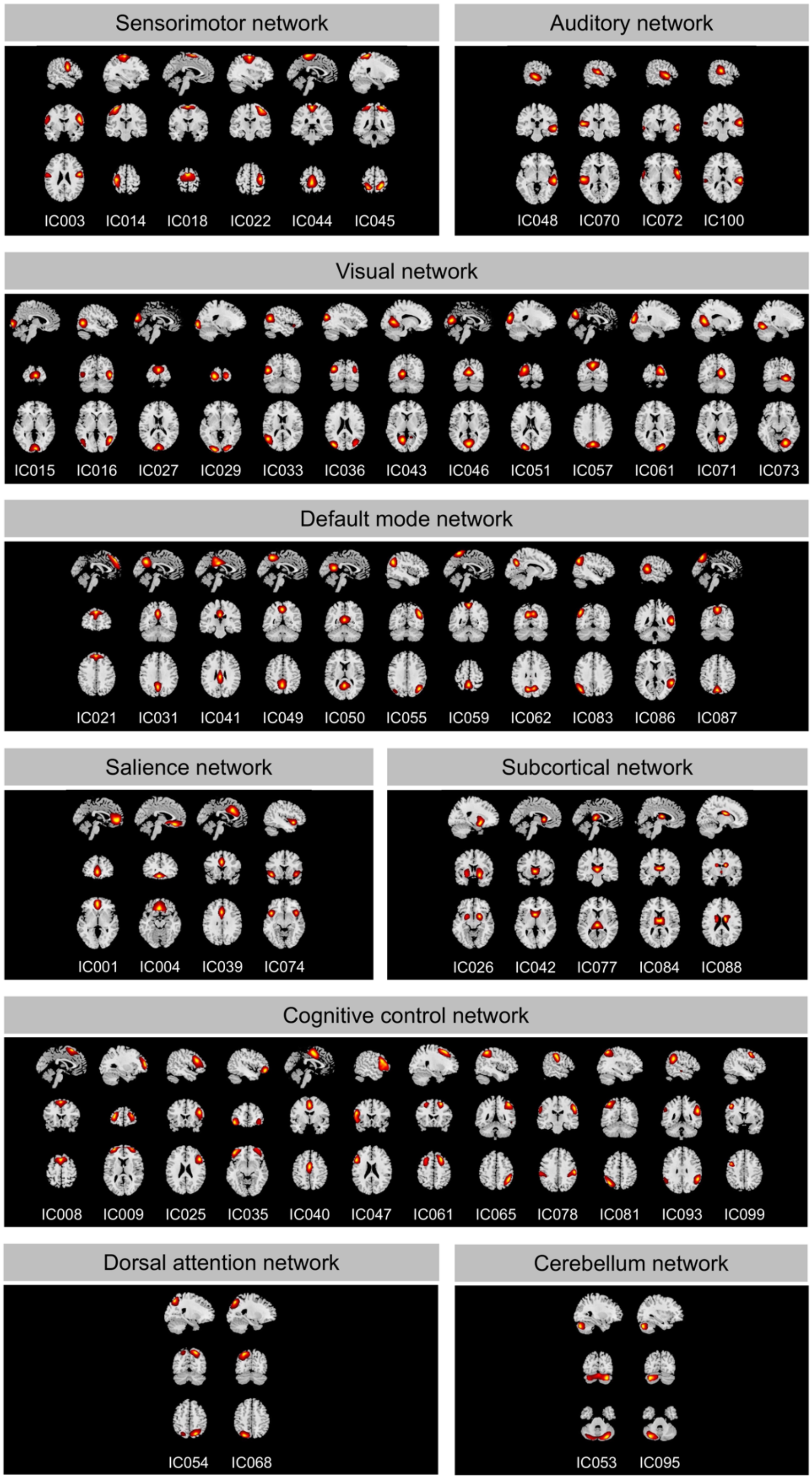

Figure S3. Spatial maps for the 59 independent components (ICs) grouped into nine functional networks (sensorimotor, auditory, visual, default mode, salience, cognitive control, dorsal attention, subcortical, and cerebellar networks). Spatial maps are displayed with threshold $t > \mu + 4\sigma$, k > 200.

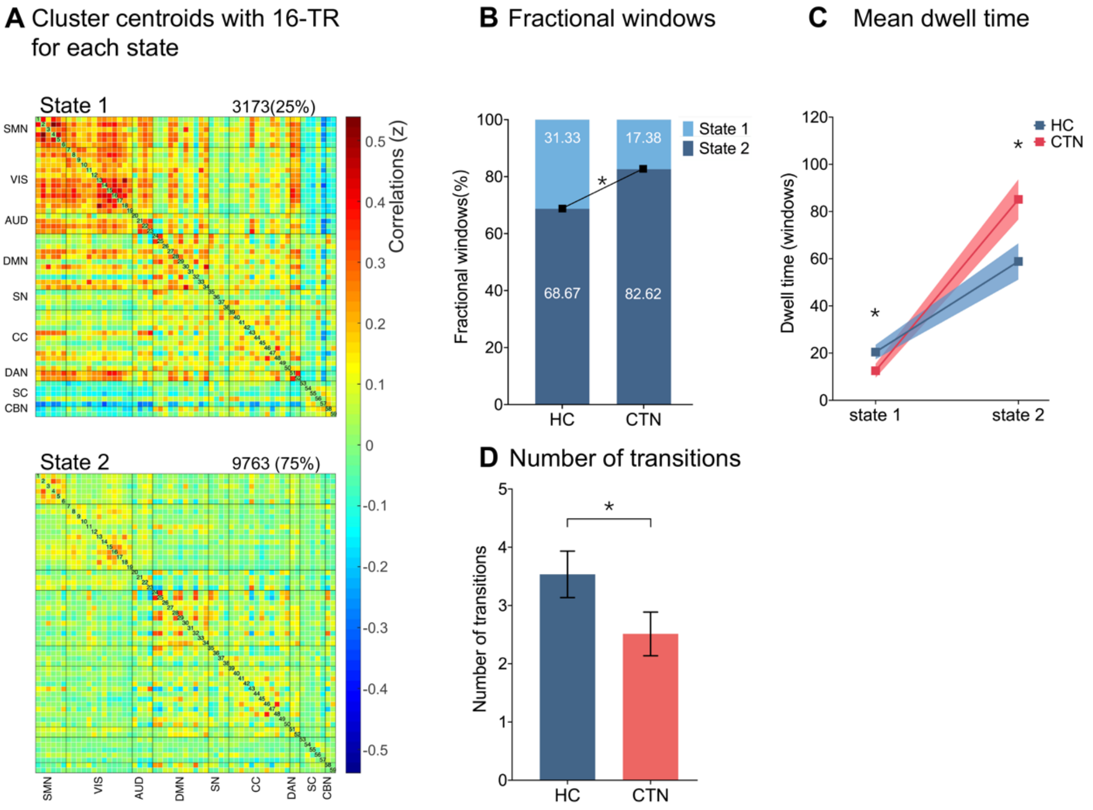

Figure S4. The validation analysis under the window size of 16-TR. (A) Cluster centroids for each state of all participants. State 1 accounted for 25%, state 2 accounted for 75%. State 1 and state 2 with 16-TR window size were similar to state 1 and state 2 in the original 20-TR analysis, respectively. (B and C) Classic trigeminal neuralgia (CTN) patients showed higher occurrence and spend more time in state 2 when compared with health controls (HC), which was converse in state 1. (D) The overall number of transitions were significantly reduced in CTN. * indicates p<0.05 (FDR correction was used for fractional windows and mean dwell time). CTN, classic trigeminal neuralgia; HC, healthy controls.

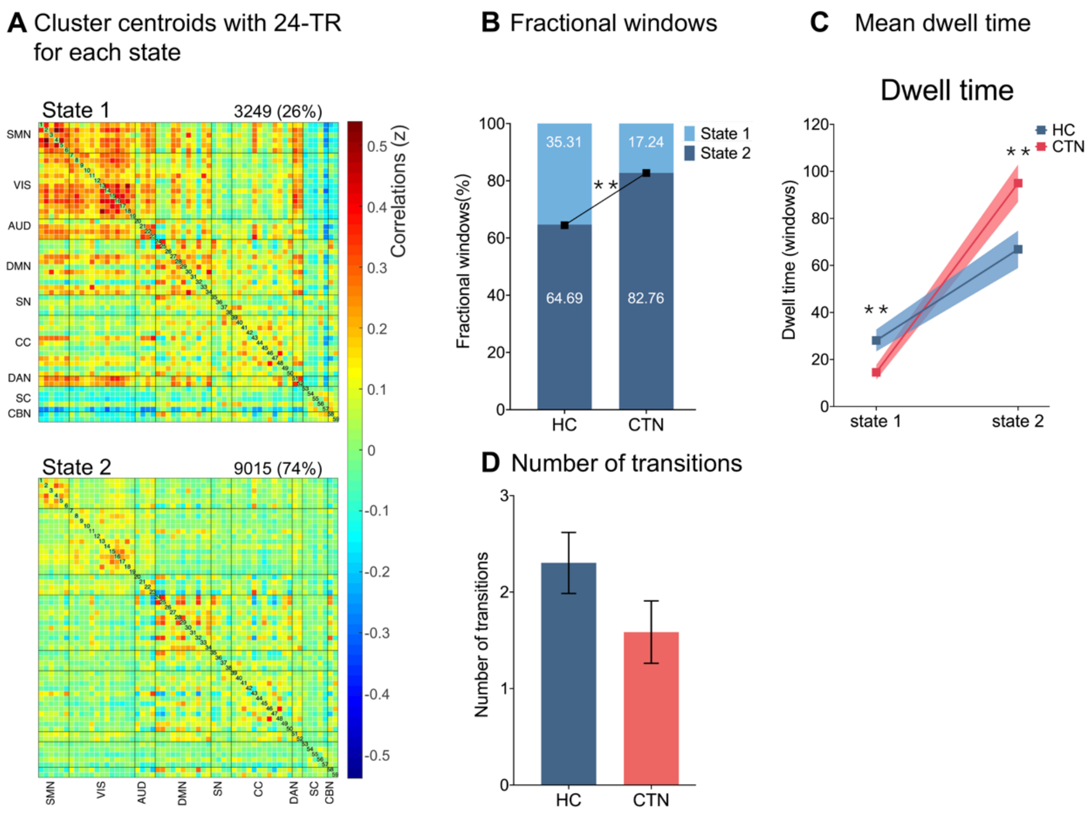

Figure S5. The validation analysis under the window size of 24-TR. (A) Cluster centroids for each state of all participants. State 1 accounted for 26%, state 2 accounted for 74%. State 1 and state 2 with 24-TR window size were similar to state 1 and state 2 in the original 20-TR analysis, respectively. (B and C) Classic trigeminal neuralgia (CTN) patients showed increased occurrence and mean dwell time in state 2 in comparison with health controls (HC), which was converse in state 1. (D) The overall number of transitions did not differ significantly between the two groups. * indicates $p<0.05$ and ** indicates $p<0.01$ (FDR correction was used for fractional windows and mean dwell time). CTN, classic trigeminal neuralgia; HC, healthy controls.

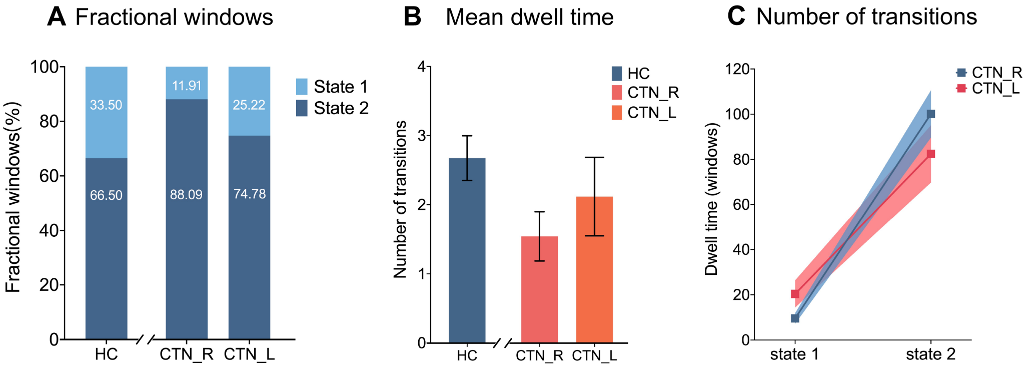

Figure S6. Subgroup analysis for temporal properties. No statistically significant differences were found for (A) the fractional windows, (B) the mean dwell time, and (C) the number of transitions between classic trigeminal neuralgia with right side pain (CTN_R) and patients with left side pain (CTN_L). CTN, classic trigeminal neuralgia; HC, healthy controls; R, right; L, left.

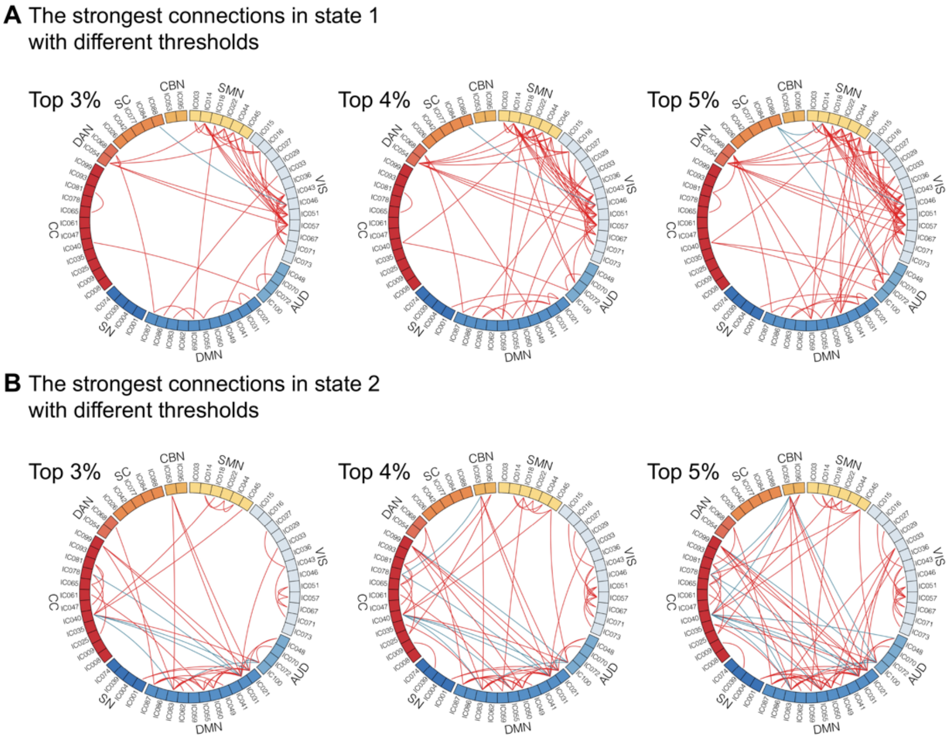

Figure S7. The strongest FC under different thresholds in each state. (A) and (B) display top FC from 3%~5% in state 1 and state 2 respectively. When using 4% and 5% as thresholds, the original connection patterns obtained by 3% were still significant

(including strong inter-connections in state1 and tight connections within DMN and CC in state 2), while many additional interfering connections also appeared and would be included in further analysis. The altered connective pattern was much clearer with 3% threshold than the other thresholds.

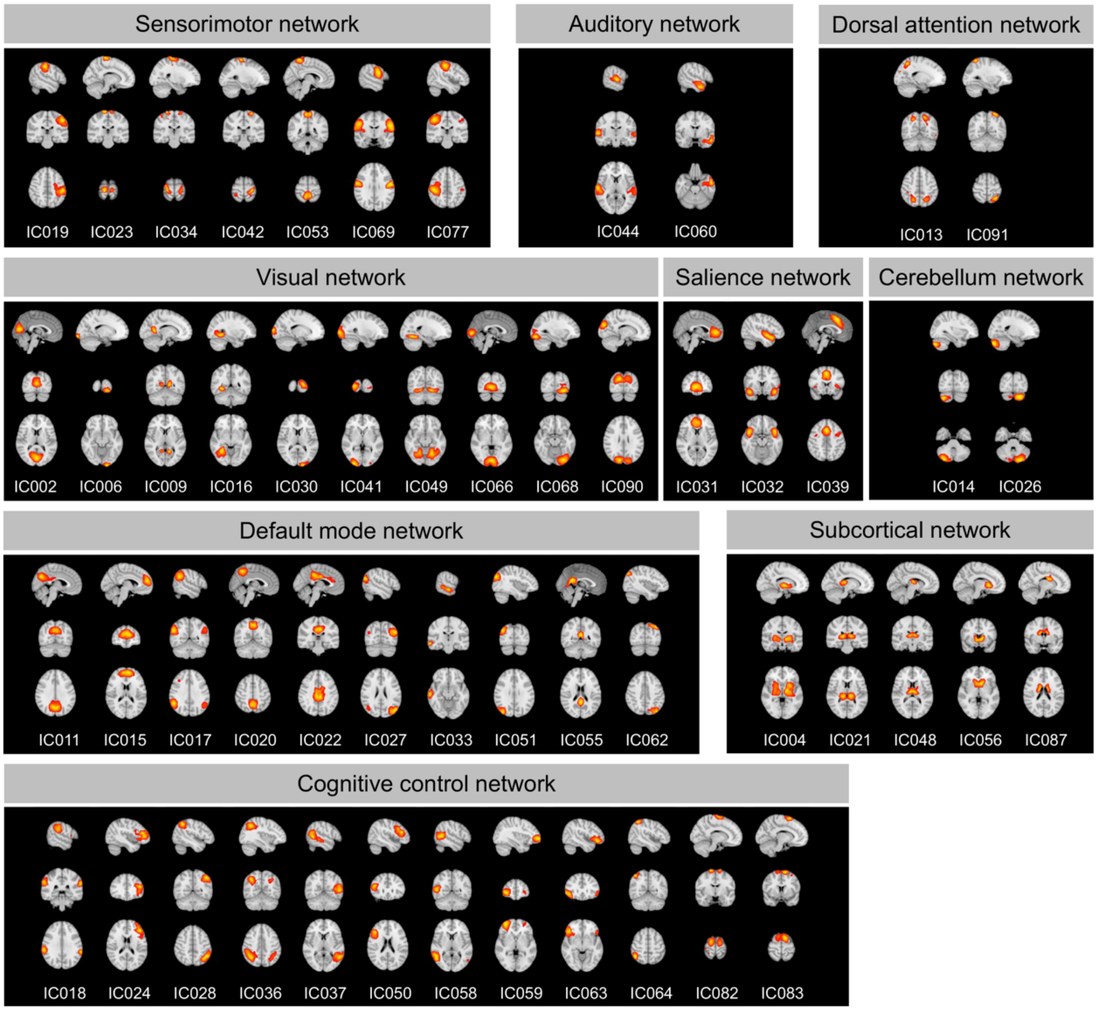

Figure S8. Spatial maps for the 53 independent components (ICs) produced by FSL. ICA analysis was performed with MELODIC of FSL using default parameters and based on same preprocessed fMRI data. ICs similar to those used in primary analysis were selected from 100 components for further dFNC analysis.

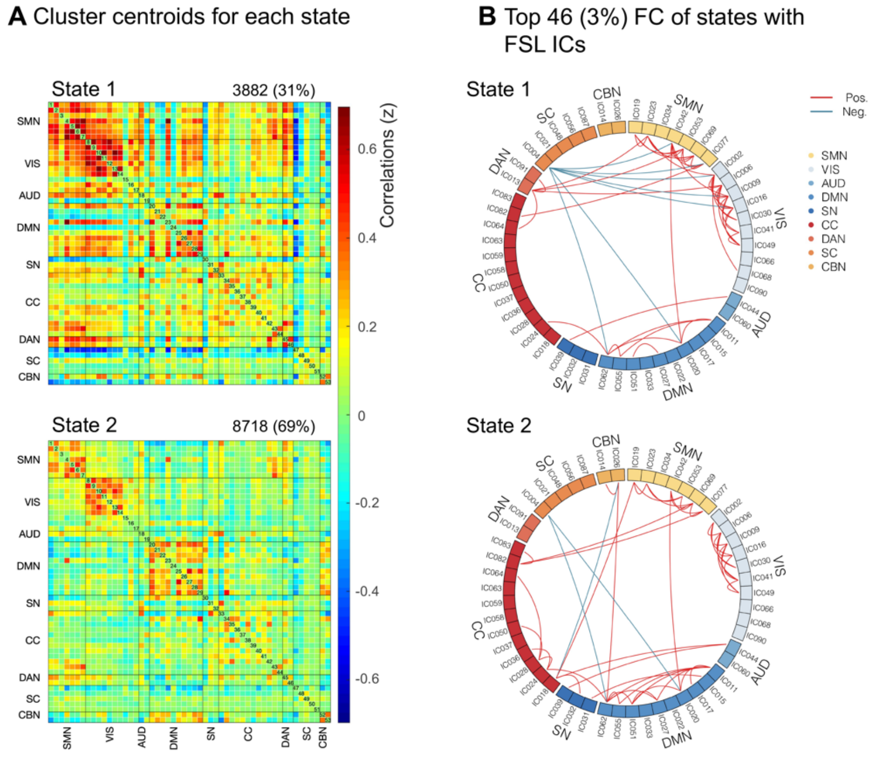

Figure S9. Clustering results in the FSL validation analysis. (A) Cluster centroids for each state. (B) The 3% strongest functional connections (absolute value) in each state are displayed. Red lines represent positive functional connectivity, while blue lines represent negative connections. The FC pattern of each state showed similarity with cluster states obtained in original analysis.

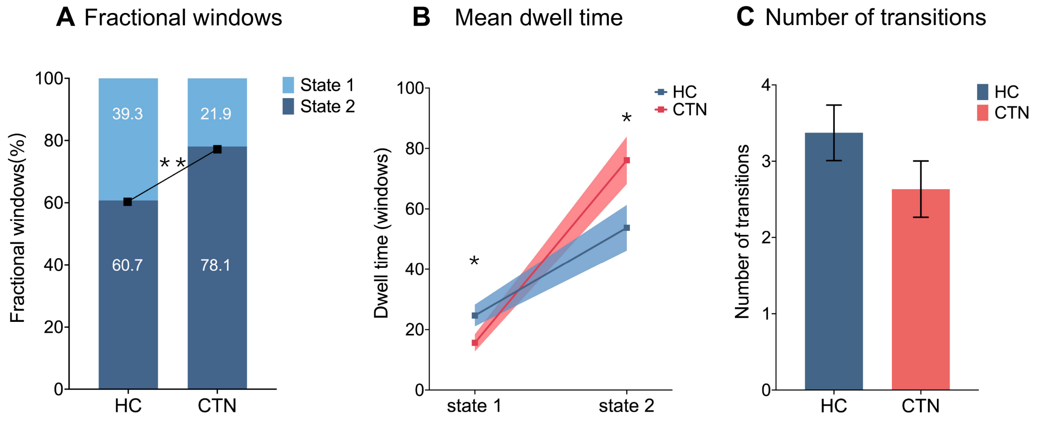

Figure S10. Results of temporal properties in the FSL validation analysis. (A) The fractional windows, (B) the mean dwell time, (C) the number of transitions are

displayed for CTN and HC. Square dots in B and bars in C reveal the mean values with shadow and error bar representing standard error. Asterisk (*) represent significance of $p<0.05$ and double asterisks (**) indicate $p<0.01$ (FDR correction was used for the fractional windows and the mean dwell time). CTN patients showed similar preference for weakly connected state 2. Though the number of transitions was reduced in patients, *p* value did not reach significant level. CTN, classic trigeminal neuralgia; HC, healthy controls.

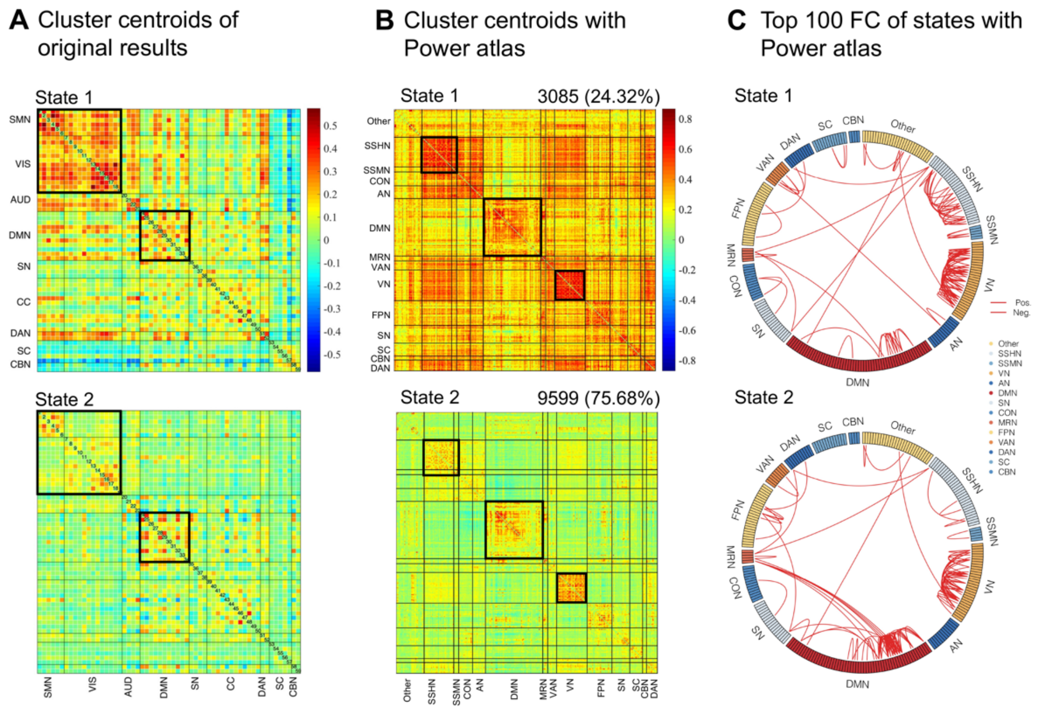

Figure S11. Clustering results in the validation analysis with Power atlas. Except the preprocessing methods used in the original study, nuisance regression (head motion parameters and white matter, cerebrospinal fluid) and smoothing (6-mm Gaussian kernel) were additionally applied. Then time-courses were extracted for each node, the sliding window approach and k-means clustering algorithm were applied with dynamic BC software (http://restfmri.net/forum/DynamicBC). (A) Original cluster centroids for

each state. (B) Cluster centroids of analysis with Power atlas. (C) The Top 100 strongest functional connections (absolute value) in each state of the validation analysis with Power atlas are displayed. Red lines represent positive functional connectivity, while blue lines represent negative connections. In the validation analysis with Power atlas, the FC pattern of each state showed similarity with original cluster states. Consistent connections within VIS, SMN and DMN are displayed with black border lines.

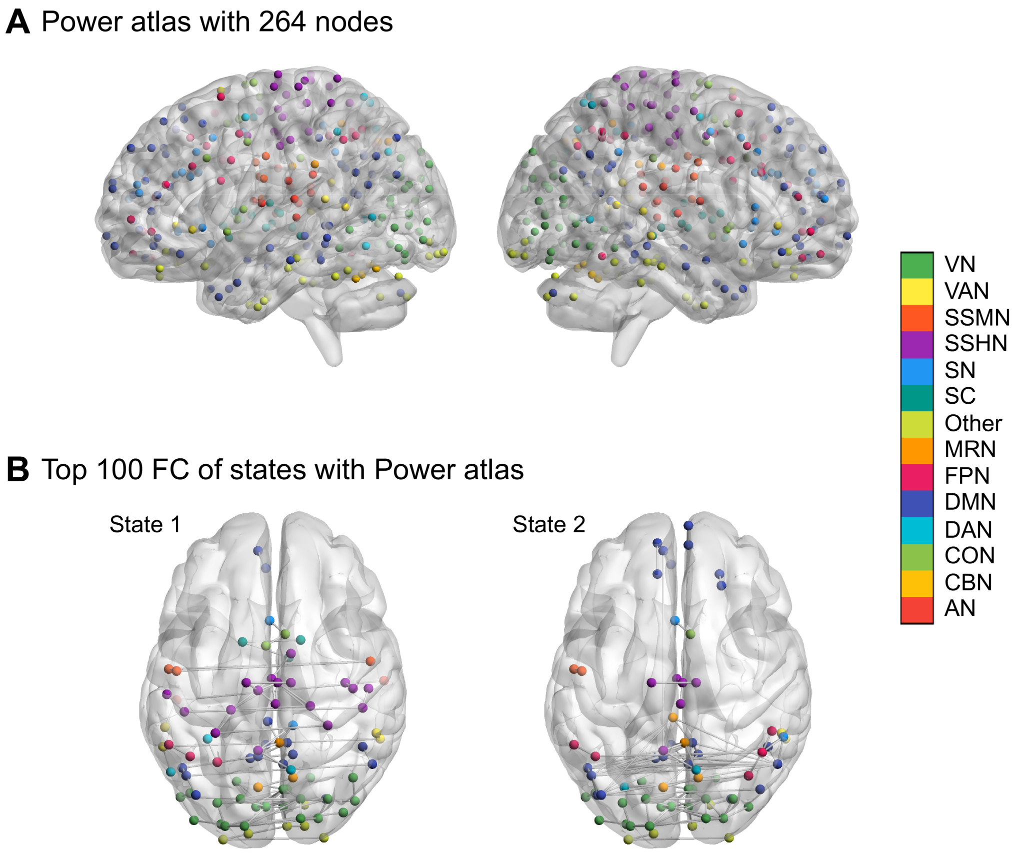

Figure S12. Distribution of nodes in Power atlas and the Top 100 connections in each state. (A) The distribution and location of 264 nodes in Power atlas. (B) The strongest connections in state 1 mainly concentrated in the perception related networks (including SMN and VIS; red, purple and dark green nodes), while in state 2, FC mainly located within DMN (blue nodes).

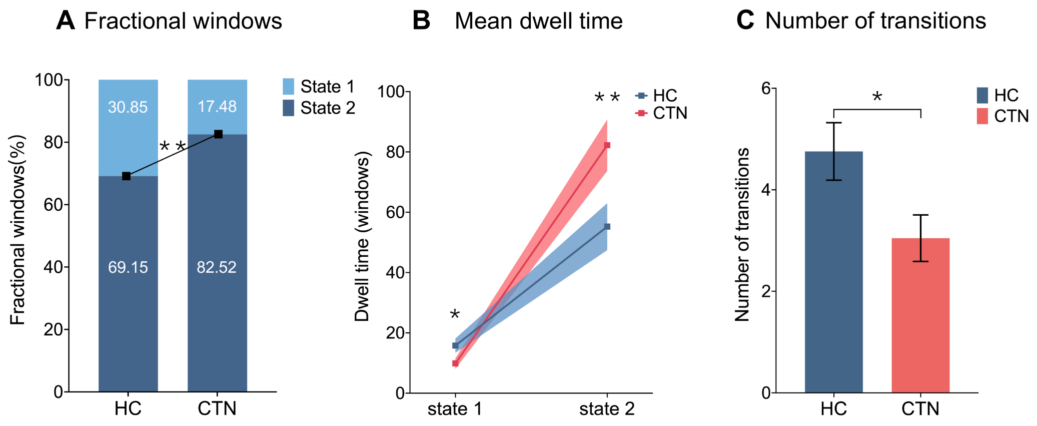

Figure S13. Results of temporal properties in the validation analysis with Power atlas. (A) The fractional windows, (B) the mean dwell time, (C) the number of transitions are displayed for CTN and HC. Square dots in B and bars in C reveal the mean values with shadow and error bar representing standard error. Asterisk (*) represents significance of $p<0.05$ and double asterisks (**) indicate $p<0.01$ (FDR correction was used for the fractional windows and the mean dwell time). Consistent differences were found in the validation analysis. CTN, classic trigeminal neuralgia; HC, healthy controls.

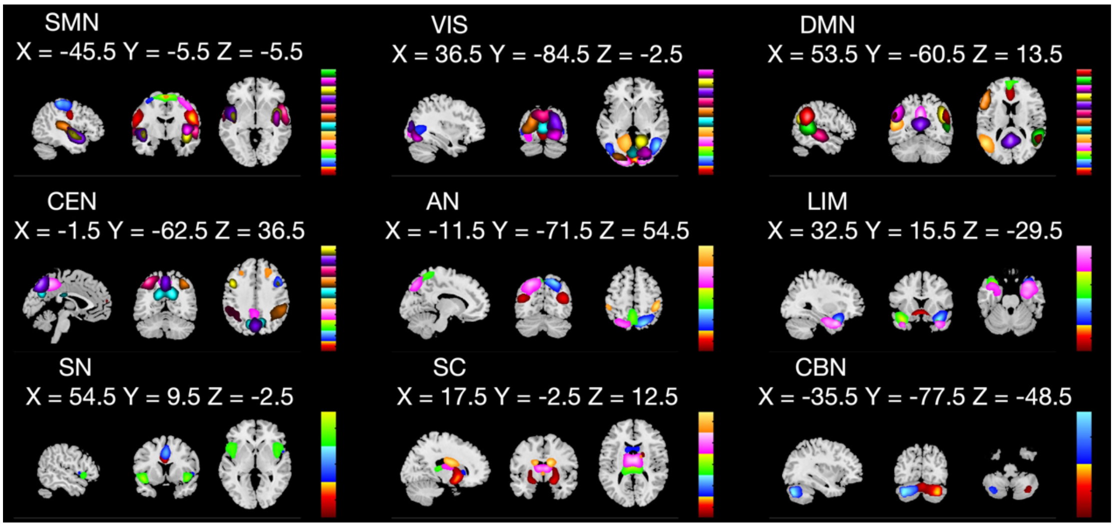

Figure S14. Spatial maps for the 65 independent components (ICs) based on Yeo atlas (7 networks). All ICs come from the 100 components used in the original analysis. ICs of AUD used before were assigned into SMN. IC001 (located in ACC) of SN used

before and IC040 (located in MCC) of CC used before were assigned into DMN and SN respectively. ICs showing similar anatomical location with Limbic network (LIM) were also selected.

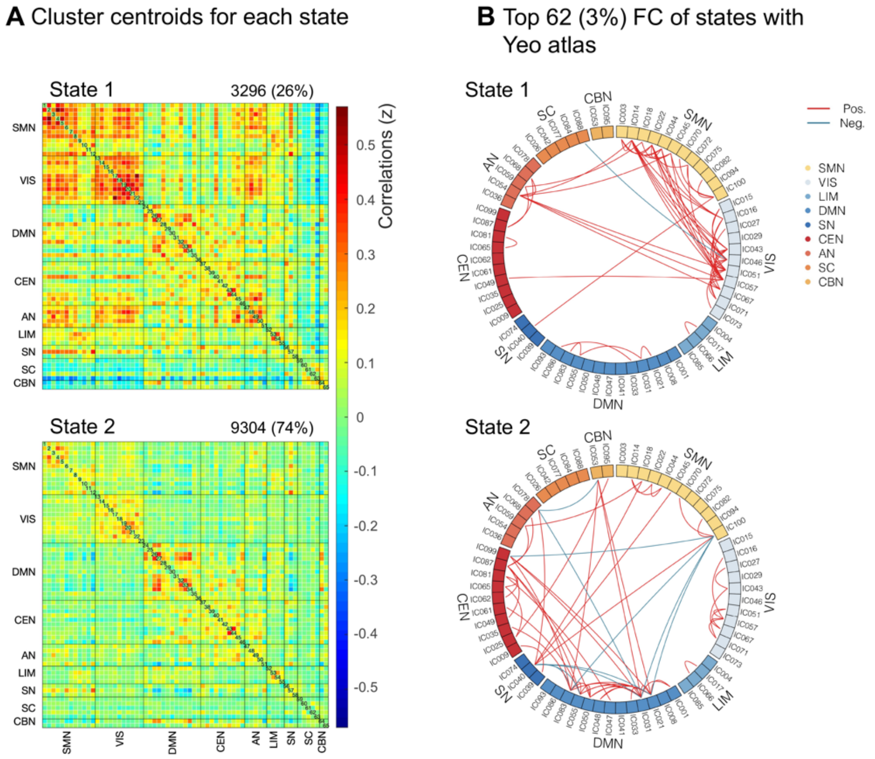

Figure S15. Clustering results in the validation analysis with Yeo atlas. (A) Cluster centroids for each state. (B) The 3% strongest functional connections (absolute value) in each state are displayed. Red lines represent positive functional connectivity, while blue lines represent negative connections. The FC pattern of each state were largely consistent with cluster states obtained in the original analysis.

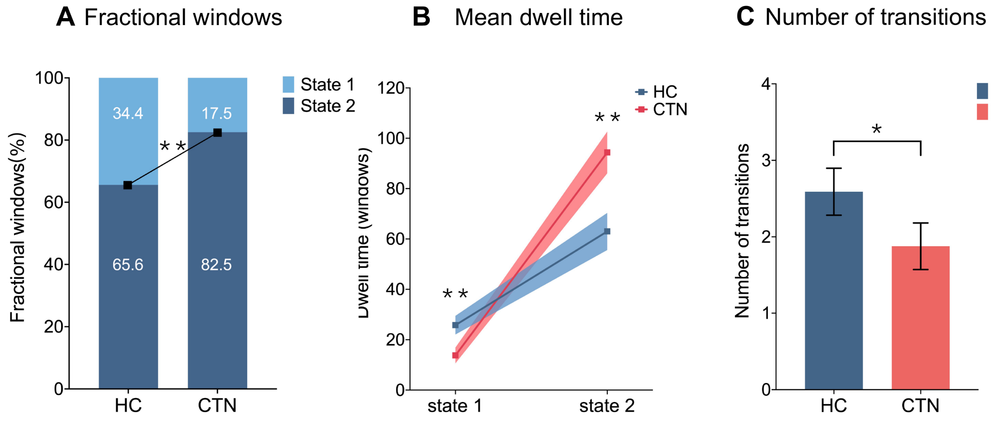

Figure S16. Results of temporal properties in the validation analysis with Yeo atlas. (A) The fractional windows, (B) the mean dwell time, (C) the number of transitions are displayed for CTN and HC. Square dots in B and bars in C reveal the mean values with shadow and error bar representing standard error. Asterisk (*) represents significance of $p<0.05$ and double asterisks (**) indicate $p<0.01$ (FDR correction was used for the fractional windows and the mean dwell time). Consistent differences were found in the validation analysis. CTN, classic trigeminal neuralgia; HC, healthy controls.

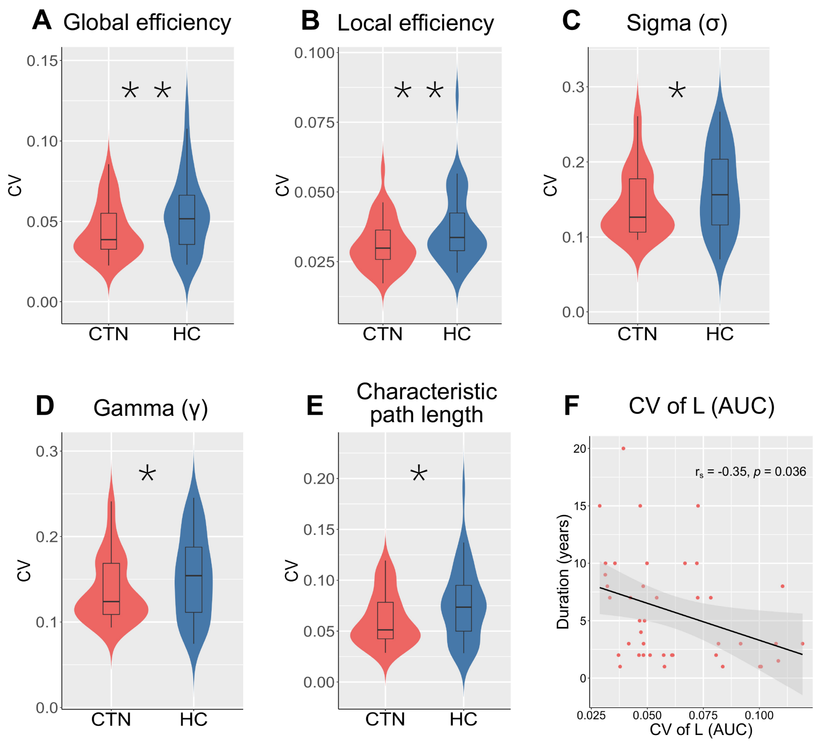

Figure S17. Dynamical topological results and correlation analysis results in the

validation analysis with Yeo atlas. Violin plots (A~E) represent differences in the CV of global efficiency, local efficiency, sigma, gamma, characteristic path length (both calculated as AUC) between CTN (red) and HC (blue). All asterisks indicate a significant group differences (*, $p<0.05$; **, $p<0.01$). Horizontal lines in boxes indicate group medians. (F) displays the negative correlation between the CV of characteristic path length (AUC) and disease duration. CV, coefficient of variation; CTN, classic trigeminal neuralgia; HC, healthy controls.

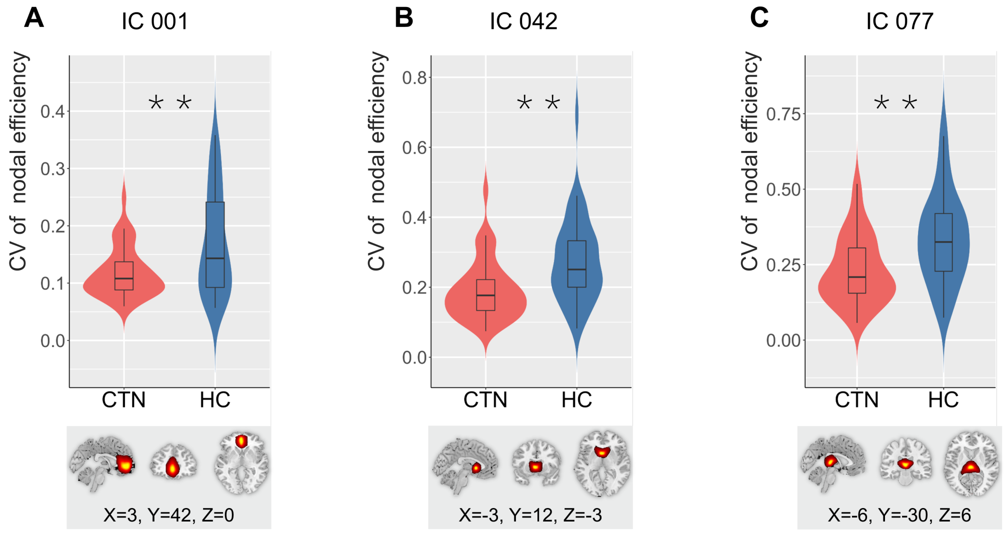

Figure S18. CV comparing of nodal efficiency (AUC) in the validation analysis with Yeo atlas. The differences were mainly located in (A) IC1 (located in ACC), (B) IC42 (located in caudate), and (C) IC77 (located in thalamus), which were consistent with original results. All asterisks indicate a significant group difference (**, $p< 0.01$, FDR corrected). Horizontal lines in boxes indicate group medians. CV, coefficient of variation; CTN, classic trigeminal neuralgia; HC, healthy controls; IC, independent component; ACC, anterior cingulate cortex.

References

1. Nour M M, Dahoun T, Mccutcheon R A et al (2019) Task-induced functional brain connectivity mediates the relationship between striatal D2/3 receptors and working memory. Elife 8. doi:10.7554/eLife.45045
2. Zheng W, Zhao Z, Zhang Z et al (2021) Developmental pattern of the cortical topology in high-functioning individuals with autism spectrum disorder. Hum Brain Mapp 42:660-675. doi:10.1002/hbm.25251
3. Shi C M, Wei B T, Wei S L et al (2021) A quantitative discriminant method of elbow point for the optimal number of clusters in clustering algorithm. Eurasip Journal on Wireless Communications and Networking 2021:16. doi:10.1186/s13638-021-01910-w
4. Kim J, Criaud M, Cho S S et al (2017) Abnormal intrinsic brain functional network dynamics in Parkinson's disease. Brain 140:2955-2967. doi:10.1093/brain/awx233
5. Fiorenzato E, Strafella A P, Kim J et al (2019) Dynamic functional connectivity changes associated with dementia in Parkinson's disease. Brain 142:2860-2872. doi:10.1093/brain/awz192
6. Latora V, Marchiori M (2001) Efficient behavior of small-world networks. Phys Rev Lett 87:198701. doi:10.1103/PhysRevLett.87.198701
7. Luo L, Li Q, You W et al (2021) Altered brain functional network dynamics in obsessive-compulsive disorder. Hum Brain Mapp 42:2061-2076. doi:10.1002/hbm.25345
8. Watts D J, Strogatz S H (1998) Collective dynamics of 'small-world' networks.

Nature 393:440-442. doi:10.1038/30918

9. Newman M E J (2003) The structure and function of complex networks. Siam Review 45:167-256. doi:10.1137/S003614450342480
